# Enabling high-mobility, ambipolar charge-transport in a DPP-benzotriazole copolymer by side-chain engineering†
†Electronic supplementary information (ESI) available: Raw materials and solvents, used equipment, synthesis routes, additional TGA, GIWAXS and T-dependent mobility data. See DOI: 10.1039/c5sc01326g Additional data related to this publication is available at https://www.repository.cam.ac.uk/


**DOI:** 10.1039/c5sc01326g

**Published:** 2015-08-12

**Authors:** Mathias Gruber, Seok-Heon Jung, Sam Schott, Deepak Venkateshvaran, Auke Jisk Kronemeijer, Jens Wenzel Andreasen, Christopher R. McNeill, Wallace W. H. Wong, Munazza Shahid, Martin Heeney, Jin-Kyun Lee, Henning Sirringhaus

**Affiliations:** a Cavendish Laboratory , University of Cambridge , J J Thomson Avenue , Cambridge , CB3 0HE , UK . Email: hs220@cam.ac.uk; b Department of Polymer Science & Engineering , Inha University , Incheon , 402-751 , South Korea . Email: jkl36@inha.ac.kr; c Department of Energy Conversion and Storage , Technical University of Denmark , Frederiksborgvej 399 , 4000 Roskilde , Denmark; d Department of Materials Engineering , Monash University , Clayton , Victoria 3800 , Australia; e School of Chemistry , Bio21 Institute , University of Melbourne , Parkville , Victoria 3010 , Australia; f Department of Chemistry and Centre for Plastic Electronics , Imperial College , London , SW7 2AZ , UK . Email: m.heeney@imperial.ac.uk

## Abstract

In this article we discuss the synthesis of four new low band-gap co-polymers based on the diketopyrrolopyrrole (DPP) and benzotriazole (BTZ) monomer unit. We demonstrate that the BTZ unit allows for additional solubilizing side-chains on the co-monomer and show that the introduction of a linear side-chain on the DPP-unit leads to an increase in thin-film order and charge-carrier mobility if a sufficiently solubilizing, branched, side chain is attached to the BTZ. We compare two different synthetic routes, direct arylation and Suzuki-polycondensation, by a direct comparison of polymers obtained *via* the two routes and show that direct arylation produces polymers with lower electrical performance which we attribute to a higher density of chain Furthermore we demonstrate that a polymer utilizing this design motif and synthesized *via* Suzuki-polycondensation (**(l-C_18_)-DPP-(b-C_17_)-BTZ**) exhibits exceptionally high and near balanced average electron and hole mobilities >2 cm^2^ V^–1^ s^–1^ which are among the highest, robustly extracted mobility values reported for DPP copolymers in a top-gate configuration to date. Our results demonstrate clearly that linear side chain substitution of the DPP unit together with co-monomers that allow for the use of sufficiently long or branched solubilizing side chains can be an attractive design motif for solution processable, high mobility DPP copolymers.

## Abstract 2

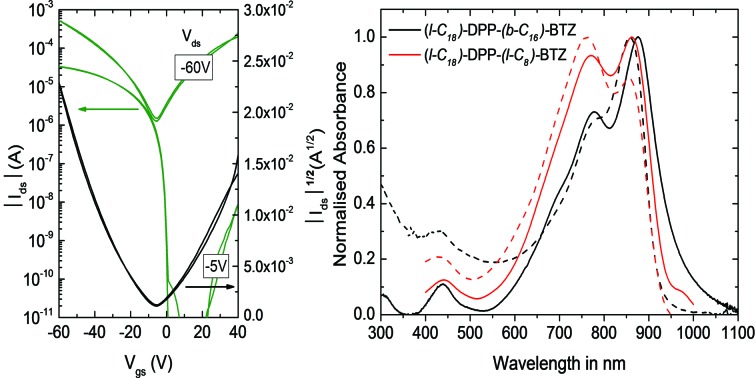
In this article we discuss the synthesis of four new low band-gap co-polymers based on the diketopyrrolopyrrole (DPP) and benzotriazole (BTZ) monomer unit.

## Introduction

Low-bandgap, donor–acceptor conjugated copolymers are being intensely researched for applications in organic field-effect-transistors (OFETs) and solar cells. They have led to the development of polymer-based OFETs with high field-effect-mobilities exceeding 1 cm^2^ V^–1^ s^–1^ which outperform amorphous-Si (a-Si:H) thin film transistors and could enable new applications for OFETs, including logic circuit applications that require fast switching and current-driven flexible displays based on organic light-emitting diodes.^1^,^2^ For logic circuit applications a complementary circuit design based on p- and n-type FETs is beneficial to enable low-power consumption and achieve adequate noise margins for larger-scale integration.^3^ Polymers that are intrinsically ambipolar, *i.e.* capable of operating in either p-type or n-type operation mode, facilitate fabrication of such circuits, either using ambipolar or genuinely complementary logic configurations. In the latter case a single polymer semiconductor is used to realize both the p-type and the n-type devices, but the device configuration is engineered to suppress either electron or hole transport, respectively, through the use of appropriate source-drain contacts or gate dielectrics.^4^ Ambipolar polymer semiconductors have reached exceptionally high performances with p- and n-type mobilities exceeding 2 cm^2^ Vs^–1^ and simple ambipolar circuits, such as ring oscillators with oscillation frequencies of up to 380 kHz and simple logic gates have been demonstrated.^4^–^6^

Some of the highest performing ambipolar donor–acceptor polymers known today are based on the diketopyrrolopyrrole (DPP) electron accepting unit. FET mobilities up to 12 cm^2^ V^–1^ s^–1^ for hole- and up to 6.3 cm^2^ V^–1^ s^–1^ for electron-transport have been claimed,^2^,^6^–^8^ although in these ambipolar materials there is ongoing debate about overestimation of mobility values, when mobilities are extracted from a narrow gate voltage range near the onset of hole accumulation, in which the hole concentration might be enhanced by residual electrons in the channel. The DPP unit is usually flanked by side units like phenylene, thiophene or selenophene. Units based on five-membered rings, such as thiophene or selenophene, tend to exhibit less backbone torsion and improved backbone co-planarity resulting in improved OFET performance compared to six-membered rings.^9^ Recently Nielsen and co-workers published a very extensive review comparing more than 80 different DPP containing polymers and summarizing the effects of different side chain substitution, co-monomers and processing conditions on FET performance.^9^ In most polymers containing the DPP unit, long, branched side-chains are attached to the DPP-core to endow the polymers with solution processability. It is generally understood that the side chain substitution exerts an important influence on the packing and backbone conformation of conjugated polymers in the solid state and may even dictate the ability of the polymer to form an ordered microstructure.^10^ Therefore choosing the right chemical type (*e.g.* alkyl, fluoroalkyl, …), structure (*e.g.* linear or branched) and length of side-chains is an important part of conjugated polymer design and can lead to major differences in electrical performance of a semiconducting polymer. The deliberate choice of side-chains to enable good electrical performance of a conjugated polymer is referred to as side-chain engineering and has gained in importance in the last few years leading to an increasing number of publications on this topic.^11^ It has been found in a number of conjugated polymers that the introduction of branched instead of linear alkyl side-chains improves polymer solubility but is often detrimental for efficient charge-transport as *e.g.* shown by Osaka *et al.* and Guo *et al.* for two series of polymers in organic field-effect transistors.^12^ In their respective works both groups found decreased charge-carrier mobilities for polymers containing branched side-chains compared to the linear side-chain substituted variants and both authors attributed the decreased transistor performance to an increase in side-chain bulkiness of branched alkyl-chains preventing efficient charge-transport through the polymer film. However by examining a series of DPP-based polymers and varying the attachment density of a branched 2-octyldodecyl side-chain Zhang *et al.* found that branched side-chains can also improve charge-carrier mobility by facilitating a favourable polymer backbone alignment in the thin film.^13^ Based on these results other groups studied the effect of branched side-chains on polymer semiconductors by evaluating the influence of branching point position, as well as side chain type in various DPP-based polymers.^14^

**Scheme 1:**
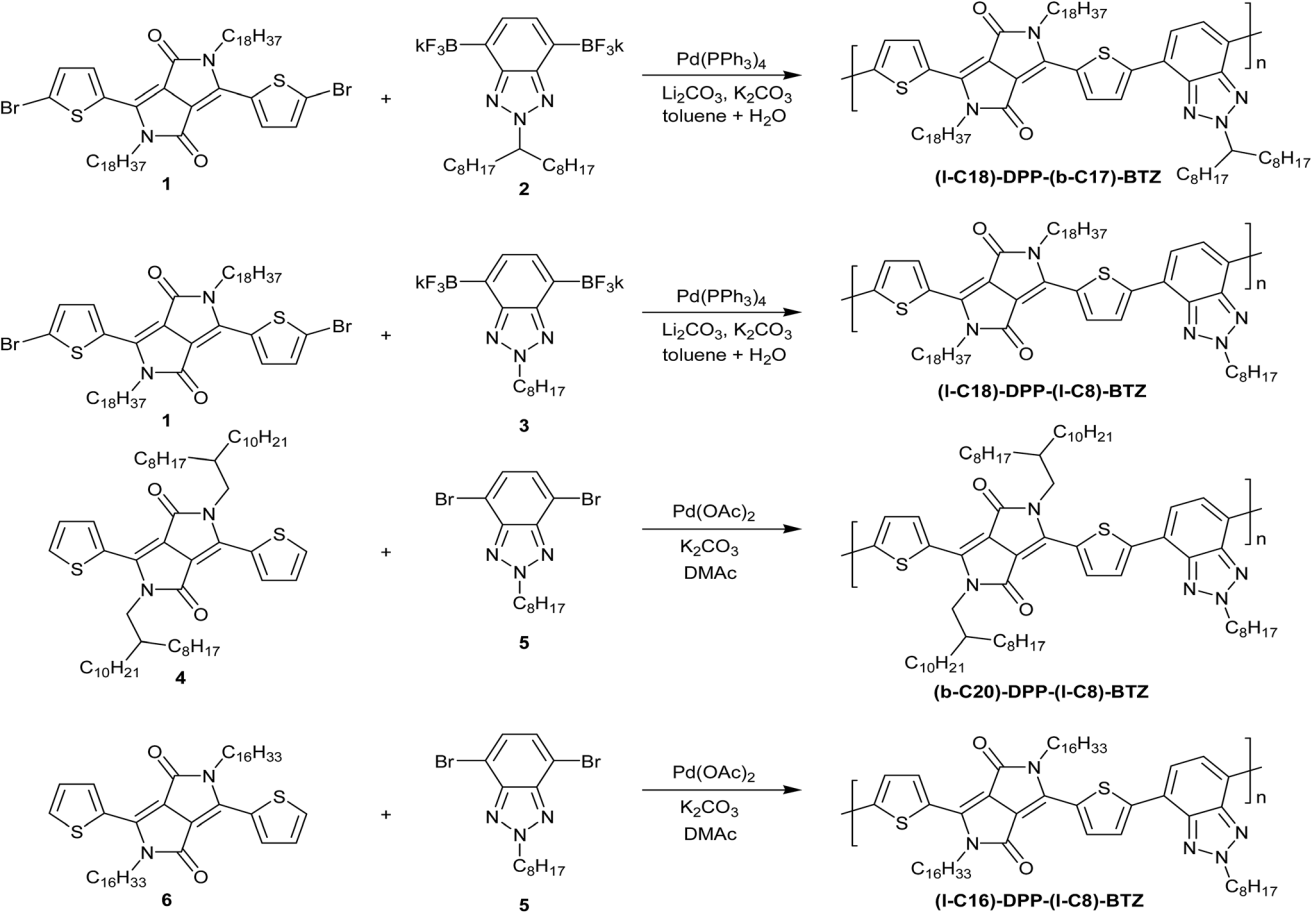
Synthesis of the four DPP-benzotriazole copolymers investigated in this study.

However despite the increasing amount of publications on this topic no universal set of rules for the efficient design of side-chains in DPP-based conjugated polymers exists up to date. Therefore further studies on the effects of side-chain substitution on the electrical properties and morphology in a wider range of DPP copolymers are urgently needed.^9^ In this publication we therefore discuss the effects of linear and branched side-chains and their positioning on thin-film morphology and electrical performance in a novel dithienyl-DPP based polymer obtained by copolymerization with the weakly electron-deficient benzotriazole (BTZ) monomers (Scheme 1). The polymer allows for facile substitution with either linear or branched side chains on both the DPP-core and the BTZ co-monomer unit. To the best of our knowledge we report in this work the first DPP-based polymer FET with a mobility >2 cm^2^ V^–1^ s^–1^ in which the DPP unit is substituted with a linear side chain. In fact, among the wide range of DPP polymers tested in our group under comparable conditions, for which mobilities were extracted in a robust manner and in a top-gate device architecture, this mobility is among the highest we have observed for this class of polymers. It is higher, for example, than the value we routinely observe for DPP-T-TT (ref. 6), one of the most widely studied and best performing DPP-copolymers.

## Experimental details

### Materials

K_2_CO_3_, Li_2_CO_3_, Pd(PPh_3_)_4_, Pd(OAc)_2_, phenylboronic acid, bromobenzene were purchased from Sigma-Aldrich or Acros and used as received. Toluene (anhydrous) and dimethylacetamide (DMAc) were received from Sigma-Aldrich and used without further purification. Monomers 2,5-di(2-octyldodecyl)-3,6-bis-(thiophenyl)-1,4-diketopyrrolo[3,4-*c*]pyrrole and 2,5-dihexadecyl-3,6-bis-(thiophenyl)-1,4-diketo-pyrrolo[3,4-*c*]pyrrole were prepared according to the literature procedures.^15^ The synthesis of the other monomers is given in the ESI.†

### Polymer synthesis and characterisation

#### **(l-C_18_)-DPP-(b-C_17_)-BTZ** polymer prepared by a Suzuki polycondensation method

To a Schlenk tube were added dipotassium (b-C_17_)-BTZ bis(trifluoroborate) **2** (0.200 g, 0.351 mmol), (l-C_18_)-DPP dibromide **1** (0.338 g, 0.351 mmol), Pd(PPh_3_)_4_ (0.02 g, 0.018 mmol), Li_2_CO_3_ (0.400 g), K_2_CO_3_ (0.400 g) and Bu_4_NBr (0.011 g, 0.035 mmol).^16^,^17^ The reaction mixture was then purged with N_2_. Toluene (N_2_ bubbled, 6 cm^3^) was added to the Schlenk tube and the resulting mixture was stirred at 90 °C for 5 min. Water (N_2_ bubbled, 4 cm^3^) was added to the tube at 90 °C. After the heterogeneous mixture was stirred for 4 h at 90 °C, a solution of phenylboronic acid (0.043 g, 0.35 mmol) dissolved in toluene (N_2_ bubbled, 2 cm^3^) was added. The reaction mixture was stirred for another 1 h at 90 °C. Bromobenzene (0.11 g, 0.70 mmol) was added to the mixture. After the reaction mixture was stirred for further 2 h, it was allowed to cool down to room temperature and precipitated into stirring MeOH (200 cm^3^). The precipitates were filtered, washed with MeOH, and dried under reduced pressure. For purification of the polymer, the dried precipitate was dissolved in CHCl_3_ (300 cm^3^) and a pipetteful amount of ammonium hydroxide was added to the solution. It was then passed through a short plug of silica gel with flushing with a copious amount of CHCl_3_ and concentrated under reduced pressure. The concentrated polymer solution was dropped into stirring MeOH (200 cm^3^). The precipitated polymer was filtered, washed in a Soxhlet extraction apparatus with acetone for 48 h and dried under reduced pressure again to give the copolymer **(l-C_18_)-DPP-(b-C_17_)-BTZ** as a dark brown solid (0.26 g).

GPC (chlorobenzene, 80 °C): *M*_n_ = 63 000 g mol^–1^, *M*_w_ = 204 000 g mol^–1^, PDI = 3.2. *T*_decomp_. (onset) = 390 °C. Found: C, 75.6; H, 10.2; N, 6.0; S, 5.5%. (C_73_H_117_N_5_S_2_O_2_)_*n*_ requires: C, 75.5; H, 10.2; N, 6.0; S, 5.5%.

#### **(l-C_18_)-DPP-(l-C_8_)-BTz** polymer prepared by a Suzuki polymerisation method

Following the procedure above (l-C_18_)-DPP dibromide **1** (0.217 g, 0.226 mmol), dipotassium(l-C_8_)-BTZ bis(trifluoroborate) **3** (0.100 g, 0.226 mmol), Pd(PPh_3_)_4_ (0.013 g, 0.011 mmol), Li_2_CO_3_ (0.200 g), K_2_CO_3_ (0.200 g), Bu_4_NBr (0.0073 g, 0.023 mmol) in toluene (3 cm^3^) were reacted at 90 °C for 5 min, after which water (2 cm^3^) was added. Following reaction for 4 h, the mixture was endcapped as above using degassed solutions of phenylboronic acid (0.028 g, 0.23 mmol) in toluene (1 cm^3^) and then bromobenzene (0.071 g, 0.45 mmol). The polymer as isolated as above to give the polymer **(l-C_18_)-DPP-(l-C_8_)-BTZ** as a dark blue solid (0.11 g):

GPC (chlorobenzene, 80 °C): *M*_n_ = 11 000 g mol^–1^, *M*_w_ = 55 000 g mol^–1^, PDI = 4.0. *T*_decomp._ (onset) = 413 °C. Found: C, 72.7; H, 9.4; N, 6.5; S, 6.1%. (C_64_H_99_N_5_S_2_O_2_)_*n*_ requires: C, 74.3; H, 9.6; N, 6.8; S, 6.2%.

#### **(b-C_20_)-DPP-(l-C_8_)-BTZ** polymer prepared by direct arylation method

A mixture of (b-C_20_)-DPP **4** (0.146 g, 0.170 mmol), (l-C_8_)-BTZ dibromide **5** (0.0661 g, 0.170 mmol), Pd(OAc)_2_ (0.0016 g, 0.007 mmol), and K_2_CO_3_ (0.0345 g, 0.25 mmol) in anhydrous DMAc (5 cm^3^) was purged with Ar stream and stirred for 72 h at 110 °C under Ar.^18^ After cooling to room temperature, the reaction mixture was precipitated into acidic MeOH and stirred for another 30 min. The resulting precipitates were filtered, washed with MeOH and purified by Soxhlet extraction with MeOH, acetone and hexane before collecting the higher molecular weight CHCl_3_ fraction. To the CHCl_3_ extract was added an aqueous solution of sodium diethyldithiocarbamate (*ca.* 1 g per 100 cm^3^) and the mixture was heated to 60 °C with vigorous stirring for 2 h. After cooling to room temperature, the layers were separated and the organic fraction was washed with water (2 × 150 cm^3^), and concentrated under reduced pressure. The resulting residue was dissolved in a minimum amount of CHCl_3_ and added dropwise to a vigorously stirred MeOH (250 cm^3^). The precipitates were filtered using a 0.45 μm PTFE filter and dried under reduced pressure to afford the desired polymer. A final purification by preparative GPC afforded the polymer as a dark powder (0.083 g, 42%).

GPC (chlorobenzene, 80 °C): *M*_n_ = 93 000 g mol^–1^*M*_w_ = 142 000 g mol^–1^, PDI = 1.5. Found: C, 74.1; H, 9.4; N, 6.1%. (C_68_H_107_N_5_S_2_O_2_)_*n*_ requires: C, 74.9; H, 9.9; N, 6.4%.

#### **(l-C_16_)-DPP-(l-C_8_)-BTZ** prepared by direct arylation method

Using a similar procedure to above, 4,7-dibromo-2-octyl-2,1,3-benzotriazole (66.1 mg, 0.17 mmol), 2,5-dihexadecyl-3,6-bis-(thiophenyl)-1,4-diketopyrrolo[3,4-*c*]pyrrole (127.4 mg, 0.17 mmol), Pd(OAc)_2_ (1.6 mg, 0.007 mmol), and K_2_CO_3_ (34.5 mg, 0.25 mmol) in anhydrous dimethylacetamide DMAc (5 cm^3^) were reacted for 72 h at 110 °C. Following purification as above l-PDPPBTz was isolated as a dark powder. Yield: 77 mg, 47%.

GPC (chlorobenzene, 80 °C): *M*_n_ = 27 600 g mol^–1^; *M*_w_ = 54 800 g mol^–1^; PDI = 2.0. Found: C, 72.9; H, 8.7; N, 6.7%. (C_60_H_89_N_5_O_2_S_2_)_*n*_ requires: C, 73.8; H, 9.2; N, 7.2%.

### Device fabrication and characterisation

Bottom-contact top-gate field-effect transistors were fabricated with poly(methyl methacrylate) (PMMA) as polymer gate dielectric. 20 nm Au source and drain contacts with a 4 nm Cr adhesion layer were constructed photolithographically on Corning 1737 alkali-free borosilicate glass substrates. The pre-structured substrates were then transferred into a nitrogen glovebox for all subsequent processing steps. All polymer semiconductor films with the exception of **(l-C_18_)-DPP-(l-C_8_)-BTZ** were deposited on top of the electrodes by spin-coating from a 9 mg mL^–1^ solution in anhydrous chlorobenzene yielding a layer thickness of 40–60 nm. As **(l-C_18_)-DPP-(l-C_8_)-BTZ** showed a very low solubility in chlorobenzene it was spin-coated from 1,2-dichlorobenzene using the same parameters as for the other polymers and yielding a comparable polymer film thickness. Afterwards the polymer films were annealed at the designated temperature for one hour before cooling to room temperature. The PMMA polymer gate dielectric film (from Polymer Source, electronic-grade, syndiotactic PMMA) was spin-coated from a 50 mg mL^–1^ solution in anhydrous *n*-butylacetate and subsequently annealed at 90 °C for 30 minutes resulting in a 500 nm thick dielectric layer. Finally Au gate electrodes were evaporated thermally through a shadow mask to complete the devices. Due to the evaporator being located in ambient atmosphere the samples were taken out of the glovebox and mounted in the evaporator in ambient air prior to evaporation.

Electrical characterization of all devices was performed with an Agilent 4155B Semiconductor Parameter Analyzer inside a nitrogen glovebox. Saturated field-effect mobilities (*μ*_sat_) were determined from the slope of *I*_D_^1/2^ over *V*_G_ in the last 10 V of the transfer curves (*μ*_h_: *V*_G_ = –50 to –60 V, *μ*_e_: *V*_G_ = 50 to 60 V) by using the following equation:*μ*_sat_ = (d*I*_D_^1/2^/d*V*_G_)^2^2*L*/(*WC*_ins_)with *L* and *W* being the transistor channel length and width and *C*_ins_ being the gate dielectric capacitance. Due to a non-negligible gate-voltage dependency of charge-carrier mobilities, extracted mobility values were slightly lower if a larger gate-voltage region was used to determine mobility. However even if almost the whole transistor operating range (*e.g.* for *μ*_h_: *V*_G_ = –60 to –30 V) was used for mobility extraction, extracted mobilities were still 70–90% of the values extracted in the last 10 V of the transfer curve and ranged up to 2 cm^2^ V^–1^ s^–1^ for the best devices. This shows that the used method for mobility extraction is robust and does not yield unrealistically high mobility values. For measurements at low temperatures a Desert Cryogenics low temperature probe station was used. Measurements at low temperatures were carried out by cooling the devices to low temperatures and reheating them afterwards. Measurement points were taken in both directions (during cooling and heating cycle).

### Thin-film structural characterization

Synchrotron-based grazing-incidence wide-angle X-ray scattering (GIWAXS) measurements of **(l-C_16_)-DPP-(l-C_8_)-BTZ** and **(b-C_20_)-DPP-(l-C_8_)-BTZ** were performed at the small/wide angle X-ray scattering beamline at the Australian Synchrotron.^19^ 14 keV photons were used with the 2D diffraction patterns recorded on an MAR-165 CCD detector. A grazing incidence angle of 0.1°, close to the films critical angle, was employed causing the X-rays to penetrate the entire film and therefore probe the films bulk structure. The sample-to-detector distance was calibrated using a silver behenate standard. Data acquisition times of 60 s were used with no evidence found for beam damage when comparing data taken at shorter and longer acquisition times. GIWAXS data were analysed using the software SAXS15ID version 3299. X-ray diffraction data are expressed as a function of the scattering vector ***Q*** that has a magnitude of 4π/*λ* sin *θ*, where *θ* is half the scattering angle and *λ* is the wavelength of the incident radiation.

Additional GIWAXS measurements of **(l-C_18_)-DPP-(b-C_17_)-BTZ** and **(l-C_18_)-DPP-(l-C_8_)-BTZ** were obtained at DTU Energy, Risø Campus using Cu K_α_ radiation (*λ* = 1.542 Å), generated by a rotating anode and focused using a 1D multilayer.^20^ The data were recorded on photostimulable image plates at a distance of 121 mm from the sample. A grazing incidence angle of 0.18° was employed, just below the critical angle of the Si wafer substrates, thus maximizing the diffracted signal from the polymer film. GIWAXS data were analysed using the software SIMDIFFRACTION.^21^

## Results and discussion

### Polymer design and synthesis

Co-polymerisation of the DPP-unit with another electron-deficient co-monomer has been explored previously to control the optical and electronic properties of such polymers. Strong acceptor units like benzothiadiazole (BT) or benzobisthiadiazole (BBT) have been used and have led to high mobility ambipolar polymer semiconductors with very low band gaps (*E*_g_ < 0.7 eV) and high ambipolar charge-carrier mobilities slightly exceeding 1 cm^2^ V^–1^ s^–1^.^22^ Despite their impressive performance, DPP polymers containing non-alkylated co-monomers are often poorly soluble even in chlorinated solvents and their solutions form gels when stored at room temperature. This constitutes important issues for their practical use. In this publication we investigate benzotriazole (BTZ) as an attractive alternative, weakly electron-deficient co-monomer unit. Compared to co-monomers like BT or BBT, BTZ offers additional solubility due to the possibility of alkylating the middle nitrogen atom. This allows us to explore the use of linear – as opposed to standard branched – side chains on the main DPP unit whilst maintaining a sufficient level of solubility and therefore enables the evaluation of the influence of side-chain type and positioning on FET performance in DPP-polymers.

We therefore synthesized four different modifications of a dithienyl-DPP-benzotriazole polymer with different linear and branched side chains on both the main DPP-core and the benzotriazole unit (see Scheme 1 for polymer chemical structures) to evaluate the influence of side-chain length, type and positioning on electrical performance. Despite the previous use of benzotriazole containing copolymers in organic photovoltaics and OFETs, to our knowledge this is the first demonstration of high performing OFETs based on DPP copolymers with a benzotriazole co-monomer.^23^

In order to access the four DPP-based polymers, we had to adopt two different synthetic protocols based on Pd catalysed cross-coupling, namely Suzuki polycondensation and direct arylation polymerisation (DARP). The first two polymers, **(l-C_18_)-DPP-(b-C_17_)-BTZ** and **(l-C_18_)-DPP-(l-C_8_)-BTZ**, were prepared by the Suzuki polycondensation reactions in which dipotassium 2-alkylbenzotriazolyl bis(trifluoroborate)s **2** and **3** were employed as stable alternatives to the boronic acid or boron ester of 2-alkylbenzotriazole.^16^ An interesting point in the synthesis of dipotassium (l-C_8_)-BTZ bis(trifluoroborate) **3** is the difficulty in transforming dibromo-2-octylbenzotriazole into the latter through a sequence of lithiation employing ^*t*^BuLi, substitution reaction with isopropoxydioxaborolane and fluorination using an aqueous KHF_2_ solution.^16^ Only a tiny amount of impure monomer **3** was recovered after recrystallization, which totally differs from the case of dipotassium (b-C_17_)-BTZ bis(trifluoroborate) **2**. It seems that the dibromo-2-octylbenzotriazole decomposes in the presence of ^*t*^BuLi. Several consecutive, failures with modified reaction conditions made us look at alternative protocols, one of which was the Suzuki–Miyaura borylation using bis(pinacolato)diboron and Pd(dppf)Cl_2_ catalyst.^24^ A sequence of borylation, conversion to the bis(trifluoroborate) **3** and crystallization from a mixture of acetonitrile and water provided the monomer **3** suitable for the synthesis of **(l-C_18_)-DPP-(l-C_8_)-BTZ**. Detailed synthetic procedures of relevant monomers are described in the ESI.†

Another important point in the synthesis of polymers is the previously reported modification of the polymerisation conditions.^16^ For the polymerisation of dibromobenzothiadiazole and dipotassium (b-C_17_)-BTZ bis(trifluoroborate) **2**, successful Suzuki polycondensations were possible employing a mixture of Et_4_NOH and LiOH. However, the same reagents were not effective for promoting condensation reactions with (l-C_18_)-DPP dibromide **1**. Only a small amount of precipitate was recovered by filtration and the solid had low molecular weight. We therefore carefully looked into the polymerisation conditions, and came to suspect an incompatibility of the hydrolysable amide functional groups in the DPP unit with the strongly basic conditions induced by Et_4_NOH and LiOH. Switching to a less basic reagent mixture of K_2_CO_3_ and Li_2_CO_3_ was thus regarded as a viable option, which allowed us to acquire the desired polymers, **(l-C_18_)-DPP-(b-C_17_)-BTZ** and **(l-C_18_)-DPP-(l-C_8_)-BTZ**.

The challenges in obtaining the boronic acid derivatives of monomer **3** discussed above prompted the investigation of alternative polymerization routes. In particular we were interested in methods which could utilize the readily available dibromo-2-octylbenzotriazole monomer **5** directly. Direct arylation polymerization (DARP) was of particular interest since organometallic coupling groups do not need to be introduced onto the DPP or BTz monomers, potentially facilitating synthesis and scale-up.^25^ However the selectivity of the polymerization when there are multiple aromatic C–H bonds present is a concern in some cases, and there have also been reports of significant homo-coupling of both monomers depending on the reaction conditions.^26^,^27^ This is a potentially significant limitation, since such defects would be chemically bound to the polymer, and could not be removed. However it is challenging to directly identify such defects in conjugated polymers, particularly with polymers which show strong aggregation in solution such as DPP based materials, due to both the low number of potential defects present and also the poor resolution of NMR based techniques for such aggregated materials. Here by preparing similar straight chain polymers, namely **(l-C_16_)-DPP-(l-C_8_)-BTZ** and **(l-C_18_)-DPP-(l-C_8_)-BTZ**, by the DARP and Suzuki routes we have an indirect method to compare the two polymerization routes, by investigating the thin film morphology and device performance of the two polymers.

Thus a co-polymer containing all linear sidechains, **(l-C_16_)-DPP-(l-C_8_)-BTZ** as well as a branched derivative **(b-C_20_)-DPP-(l-C_8_)-BTZ** in which the branching sidechain was on the DPP unit were prepared. Both polymers were obtained using the phosphine free conditions described by Scherf and co-workers utilizing Pd(OAc)_2_ as a catalyst with potassium carbonate in DMAc.^28^ Following purification by solvent extraction and preparative GPC, the two polymers were isolated in yields of ∼40%.

The molecular weights of all four polymers were measured by gel permeation chromatography in chlorobenzene at 80 °C against polystyrene standards, and the results are shown in Table 1. All four polymers show relatively high average molecular weights (*M*_w_) with broadly comparable values obtained for both polymerization methods. It is notable that both sets of branched polymers have considerably higher molecular weight than the linear polymers made by the same polymerization chemistry, which we ascribe to the improved solubility of the branched sidechain polymers. This helps to keep the growing polymer chain in solution during the polymerization process. Attempts to characterize the polymers by ^1^H NMR were not very informative due to the broad, poorly resolved peaks observed. This is a common issue for relatively rigid low band gap polymers which show pronounced aggregation in solution.

**Table 1:** Properties of the synthesized polymers. Number and weight average molecular weight (*M*_n_/*M*_w_), polydispersity index (PDI), maximum absorption wavelength in chlorobenzene solution and in thin-films (*λ*_max,solution_/*λ*_max,film_) estimated optical band gap (*E*_g_) and average extracted hole and electron mobility in saturation at optimized film annealing temperature (*μ*_h_/*μ*_e_)

	*M*_n_/*M*_w_^*a*^ [kDa]	PDI	*λ*_max,solution_ [nm]	*λ*_max,film_ [nm]	*E*_g_ ^*b*^ [eV]	*μ*_h_ [cm^2^ V^–1^ s^–1^]	*μ*_e_ [cm^2^ V^–1^ s^–1^]	*I*_on_/*I*_off_
**(l-C_18_)-DPP-(b-C_17_)-BTZ**	63/204	3.2	858	875	1.25	2.4	1.5	>10^7^
**(l-C_18_)-DPP-(l-C_8_)-BTZ**	11/55	4.0	764	860	1.32	0.48	0.31	>10^5^
**(l-C_16_)-DPP-(l-C_8_)-BTZ**	28/55	2.0	777	780	1.28	0.075	0.073	>10^5^
**(b-C_20_)-DPP-(l-C_8_)-BTZ**	93/142	1.5	836	840	1.30	0.011	0.018	>10^4^

^*a*^Determined by GPC.

^*b*^Determined from onset of optical absorption.

### Polymer properties

**Fig. 1:**
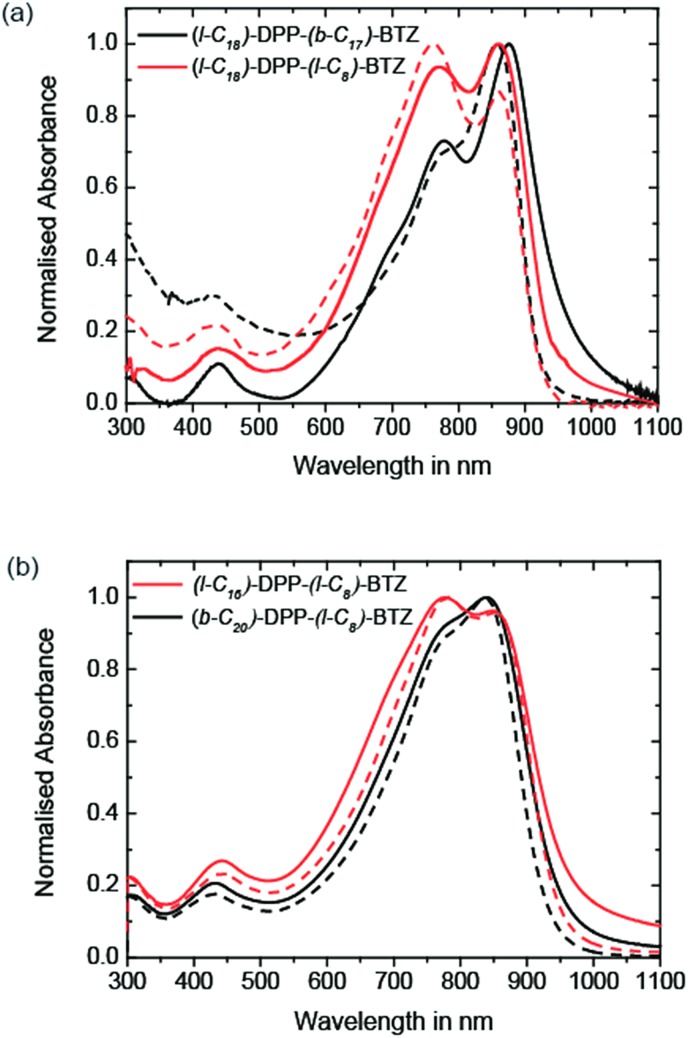
(a) UV-VIS absorption spectra of **(l-C_18_)-DPP-(b-C_17_)-BTZ** (black) and **(l-C_18_)-DPP-(l-C_8_)-BTZ** (red) measured in chlorobenzene solution (dashed lines) and thin-films (solid lines). (b) UV-VIS absorption spectra of **(l-C_16_)-DPP-(l-C_8_)-BTZ** (red) and **(b-C_20_)-DPP-(l-C_8_)-BTZ** (black) measured in chlorobenzene solution (dashed lines) and thin-films (solid lines).

The UV-VIS absorption characteristics of the different DPP-BTZ variations in thin-film and in chlorobenzene solution are shown in Fig. 1. The band gaps of all polymers described in this work are estimated from the onset of the low energy absorption peak in the solid state and are shown together with other physical data in Table 1.

All four polymers exhibit a double peak in their absorption spectra commonly observed in many DPP based polymers and a broad absorption band ranging from 500 up to 1040 nm.^2^,^4^

A comparison between **(l-C_16_)-DPP-(l-C_8_)-BTZ** and **(b-C_20_)-DPP-(l-C_8_)-BTZ** shows that the replacement of a branched side chain at the DPP unit with a longer linear side chain results in a major change in absorption behaviour. The replacement leads to an increase in intensity of the 0-1 absorption peak at 777 nm while the 0-0 absorption peak shifts from 840 to 849 nm and decreases in intensity. It also leads to a slightly lower band gap of **(l-C_16_)-DPP-(l-C_8_)-BTZ** (*E*_g_ = 1.28 eV) compared to **(b-C_20_)-DPP-(l-C_8_)-BTZ** (*E*_g_ = 1.30 eV) which is indicative of a longer conjugation length. In both polymers the solution and thin film spectra look alike suggesting similar polymer conformations in the solution and solid states.

Comparing the two linear sidechain polymers made by different polymerisation routes **(l-C_18_)-DPP-(l-C_8_)-BTZ** and **(l-C_16_)-DPP-(l-C_8_)-BTZ**, both polymers show similar positions of the two main peaks in solution (861 and 761 nm) and in thin-films (860 and 770 nm). However, we detect a change in the onset of absorption with the **(l-C_16_)-DPP-(l-C_8_)-BTZ** polymer prepared by direct arylation exhibiting a less well defined transition and a smaller band gap of 1.28 eV compared to 1.32 eV for the **(l-C_18_)-DPP-(l-C_8_)-BTZ** polymer prepared by Suzuki polycondensation. These changes are similar to those recently identified by Janssen *et al.* and are most likely the result of homo-coupling defects during the DARP polymerization.^29^ Such homo couplings would likely act as traps.

The most interesting behaviour is exhibited by **(l-C_18_)-DPP-(b-C_17_)-BTZ**, which has a branched side-chain attached to the BTZ unit together with a slightly longer linear side-chain on the DPP unit. It exhibits the best resolved fine structure of the absorption bands and the largest 0-0/0-1 peak ratio indicating a small reorganisation energy. Together with the small band gap value of 1.25 eV and a considerable red shift of the main absorption maximum from 858 to 875 nm upon thin-film formation this suggests that the most planar, ordered backbone conformation and the longest conjugation length among the four polymers is present in **(l-C_18_)-DPP-(b-C_17_)-BTZ**. This is fully consistent with the FET performance reported below.

### Thin-film microstructure

**Fig. 2:**
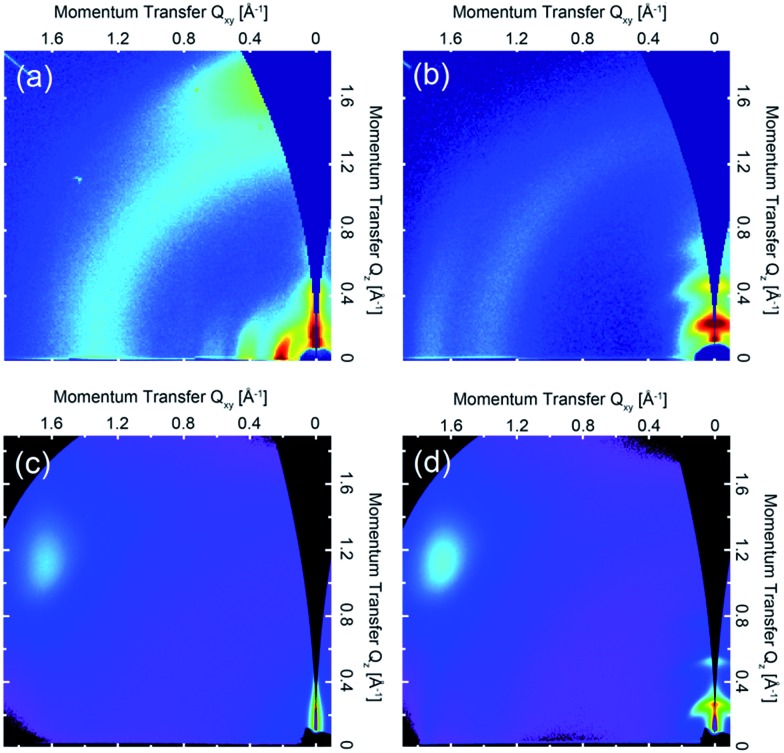
2D GIWAXS patterns of (a) **(l-C_18_)-DPP-(b-C_17_)-BTZ** film annealed at 110 °C, (b) **(l-C_18_)-DPP-(l-C_8_)-BTZ** film annealed at 110 °C, (c) **(b-C_20_)-DPP-(l-C_8_)-BTZ** annealed at 200 °C and (d) **(l-C_16_)-DPP-(l-C_8_)-BTZ** also annealed at 200 °C. Intensities are shown on a false color log scale.

**Fig. 3:**
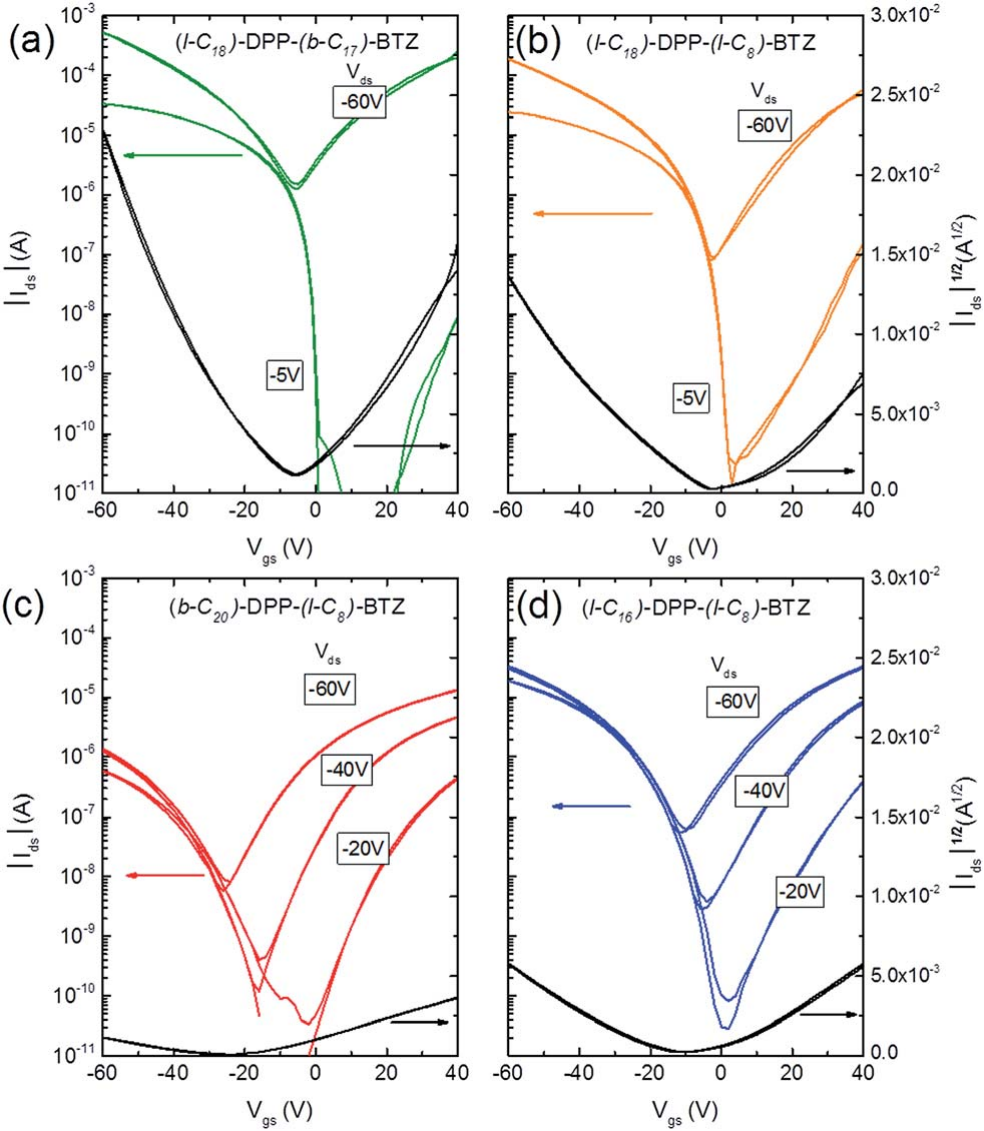
Transfer characteristics of ambipolar transistors (*L* = 20 μm, *W* = 1000 μm) based on (a) **(l-C_18_)-DPP-(b-C_17_)-BTZ** annealed at 110 °C, (b) **(l-C_18_)-DPP-(l-C_8_)-BTZ** annealed at 110 °C, (c) **(b-C_20_)-DPP-(l-C_8_)-BTZ** annealed at 200 °C and (d) **(l-C_16_)-DPP-(l-C_8_)-BTZ** annealed at 200 °C.

The 2D GIWAXS patterns of films of all four polymers annealed at temperatures to achieve optimum charge-transport are presented in Fig. 2. The scattering pattern of **(b-C_20_)-DPP-(l-C_8_)-BTZ** (see Fig. 2c) is essentially featureless indicating a lack of long-range periodic order and a near amorphous microstructure. The ring at ***Q*** ∼ 0.38 Å^–1^ is due to scattering from a Kapton window used in the experiment. In contrast, films of **(l-C_16_)-DPP-(l-C_8_)-BTZ** (see Fig. 2d) exhibit a series of (h00) scattering peaks oriented along ***Q***_*z*_ corresponding to a semicrystalline microstructure with edge-on orientated lamellae. The (100) peak is located at ***Q***_*z*_ ∼ 0.27 Å^–1^ corresponding to a lamellar stacking distance of ∼23.2 Å. A faint (010) stacking peak is observed at ***Q***_*xy*_ ∼ 1.75 Å^–1^, corresponding to a π–π stacking distance of ∼3.6 Å. GIWAXS measurements of **(l-C_18_)-DPP-(l-C_8_)-BTZ** (see Fig. 3b) show a comparable semicrystalline thin-film structure as **(l-C_16_)-DPP-(l-C_8_)-BTZ** with the first three orders of the (h00) scattering peaks along the ***Q***_*z*_-axis and a π–π stacking peak at ***Q***_*xy*_ ∼ 1.75 Å^–1^. The position of the (100) peak at ***Q***_*z*_ ∼ 0.24 Å^–1^ corresponds to a lamellar stacking distance of ∼25.7 Å. This slightly longer stacking distance is consistent with the difference in side-chain length between the two polymers. However **(l-C_18_)-DPP-(l-C_8_)-BTZ** is more ordered as three diffraction orders along ***Q***_*z*_ are clearly visible compared to two peaks in **(l-C_16_)-DPP-(l-C_8_)-BTZ** with a weak indication of the 3^rd^ order peak. Furthermore, the pi-stacking peak is better defined for this polymer. The ring at 1.4 Å^–1^ is due to packing of disordered side-chains.

Obviously films of **(l-C_16_)-DPP-(l-C_8_)-BTZ** and of **(l-C_18_)-DPP-(l-C_8_)-BTZ** which both incorporate a linear side-chain on the DPP-unit are more ordered than films of the **(b-C_20_)-DPP-(l-C_8_)-BTZ** polymer with a branched side-chain on the DPP unit. This is not entirely unexpected and is consistent with results from Tamayo *et al.* who studied film-order in films formed of DPP molecules and observed a similar increase in film disorder when incorporating branched side-chains at the DPP-core.^30^ This is likely because linear side-chains on the DPP-unit do not interfere with aggregation as much as branched side-chains which provide a higher level of steric hindrance.

A different behaviour is observed when the branched side-chain is not incorporated on the DPP-unit but on the BTZ unit instead. As the GIWAXS data in Fig. 2a clearly shows, thin-films of **(l-C_18_)-DPP-(b-C_17_)-BTZ** are highly ordered. They exhibit clear semicrystalline order but with a different texture as evidenced by the occurrence of the h00 peaks along the ***Q***_*xy*_ direction, *i.e.* a face-on orientation with the π-stacking peak centred along the ***Q***_*z*_ axis (surface normal). The 100 peak is located at ***Q***_*xy*_ ∼ 0.22 Å^–1^, corresponding to a lamellar packing distance of ∼28.6 Å, and second and third order diffraction peaks at higher ***Q***_*xy*_ values. Peaks at the same positions are present in the out-of-plane direction as well but show reduced intensity compared to the in-plane peaks. This likely corresponds to a minority component of edge-on oriented crystallites. The weak ring at *q* ∼ 1.3 Å^–1^ (∼4.8 Å) is most likely caused by packing of disordered side-chains. These results indicate a distribution of face-on and edge-on oriented crystallites with a preferred face-on crystal orientation and a high overall degree of out-of-plane π–π stacking with short (∼3.7 Å) π–π stacking distance.

Obviously the positioning of a long branched side-chain at the BTZ unit in comparison to a linear side-chain leads to a significant enhancement in thin-film order. This is especially surprising as the comparison of **(l-C_16_)-DPP-(l-C_8_)-BTZ** and **(b-C_20_)-DPP-(l-C_8_)-BTZ** shows that a branched side-chain at the main DPP-core significantly hinders the formation of an ordered film in comparison to a linear side-chain. The strong tendency of the DPP-units to form aggregated structures is widely known and was already shown for oligothiophene DPP small molecules by Tamayo *et al.* who demonstrated that the DPP units stack in a coplanar fashion and form columnar stacks.^30^ It is not unreasonable to assume that the same stacking mechanism of DPP-units is present in DPP-containing polymers but can be hindered by bulky side-chains. Clearly, the addition of a branched side-chain at the BTZ unit not only improves solubility but also does not hinder stacking of DPP units which is evident from the high degree of π–π stacking present in thin-films of **(l-C_18_)-DPP-(b-C_17_)-BTZ**. Furthermore it is interesting to note that a high degree of thin-film order is already present at low annealing temperatures of 110 °C and can be further improved using higher annealing temperatures up to 300 °C (see Fig. S3†).

### Charge-transport and ambipolar transistors

**Fig. 4:**
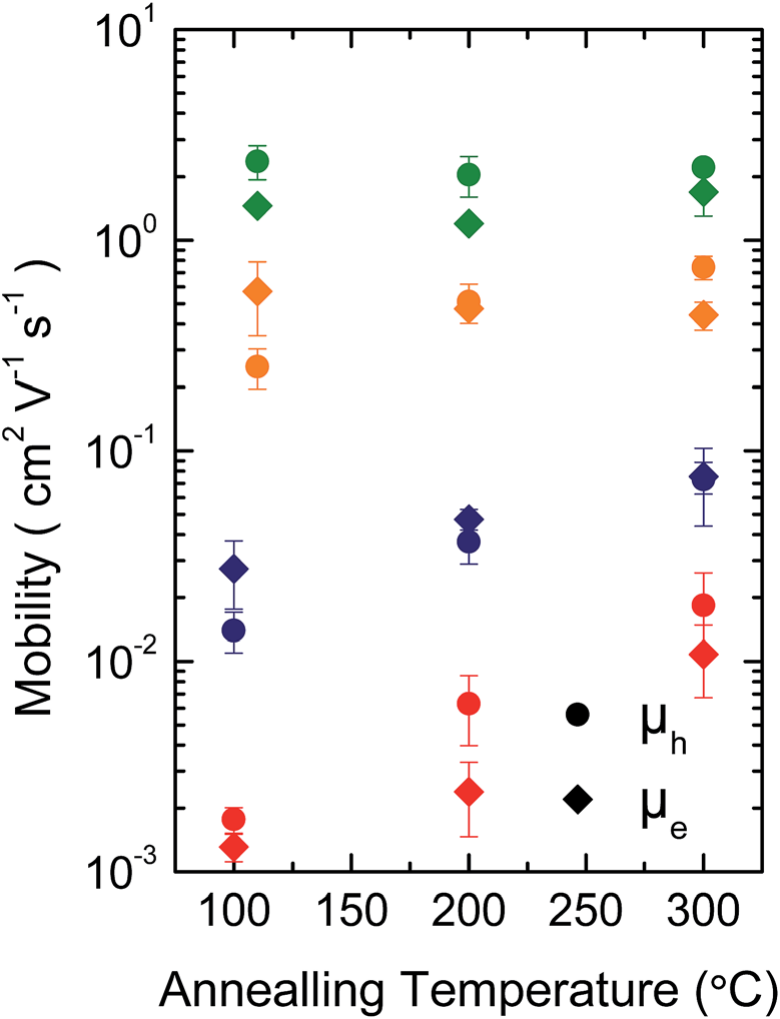
Temperature dependence of saturated hole (circles) and electron (diamonds) mobility of **(l-C_18_)-DPP-(b-C_17_)-BTZ** (green), **(l-C_18_)-DPP-(l-C_8_)-BTZ** (orange), **(l-C_16_)-DPP-(l-C_8_)-BTZ** (blue) and **(b-C_20_)-DPP-(l-C_8_)-BTZ** (red).

Fig. 3 shows representative transfer characteristics of bottom-contact top-gated field-effect transistors based on the four versions of DPP-BTZ polymer. As dielectric a 500 nm thick PMMA layer was used to prevent electron trapping at the semiconductor–dielectric interface and therefore enable n-type or ambipolar charge-transport.^4^,^6^,^31^ All transistors exhibit ambipolar transfer characteristics with low hysteresis and in the case of **(l-C_16_)-DPP-(l-C_8_)-BTZ** even balanced electron and hole transport. The extracted saturated hole- and electron-mobilities of all four polymers as a function of temperature are shown in Fig. 4.

In general **(l-C_16_)-DPP-(l-C_8_)-BTZ** and **(b-C_20_)-DPP-(l-C_8_)-BTZ** which were synthesized by direct arylation show lower transistor performances compared to **(l-C_18_)-DPP-(b-C_17_)-BTZ** and **(l-C_18_)-DPP-(l-C_8_)-BTZ** which were synthesized by Suzuki polycondensation. Both polymers exhibit a similar trend of higher annealing temperatures resulting in increased saturated field-effect mobilities.

The maximum extracted values for hole and electron mobilities were obtained after annealing at 300 °C and amount to 0.075 and 0.073 cm^2^ V^–1^ s^–1^ for **(l-C_16_)-DPP-(l-C_8_)-BTZ** and 0.011 and 0.018 cm^2^ V^–1^ s^–1^ for **(b-C_20_)-DPP-(l-C_8_)-BTZ** respectively. The almost seven-fold increase of charge carrier mobilities from **(b-C_20_)-DPP-(l-C_8_)-BTZ** to **(l-C_16_)-DPP-(l-C_8_)-BTZ** is consistent with the increase in thin-film order as observed in the GIWAXS data. The linear side-chains used for **(l-C_16_)-DPP-(l-C_8_)-BTZ** lead to a better π–π stacking and improved charge-transport.

As **(l-C_16_)-DPP-(l-C_8_)-BTZ** and **(l-C_18_)-DPP-(l-C_8_)-BTZ** are almost identical in terms of their chemical structure and mainly differ in terms of synthesis method (DARP *vs.* Suzuki polycondensation), molecular weight (see Table 1) and a small difference in side-chain length, it is interesting to draw a direct comparison between the transistor performance of the two polymers. **(l-C_16_)-DPP-(l-C_8_)-BTZ** has a slightly higher molecular weight than **(l-C_18_)-DPP-(l-C_8_)-BTZ** but exhibits lower saturated electron and hole mobilities of 0.075 cm^2^ V^–1^ s^–1^ and 0.073 cm^2^ V^–1^ s^–1^ compared to 0.48 cm^2^ V^–1^ s^–1^ and 0.31 cm^2^ V^–1^ s^–1^ for **(l-C_18_)-DPP-(lC_8_)-BTZ**. Since in most polymer systems mobility is found to increase with molecular weight rather than decrease, the difference in device performance between these two polymers is most likely a reflection of the different synthesis routes used. The direct arylation method has been reported to incorporate homo-coupling defects into the chain, and such defects are expected to affect device performance adversely.^27^,^29^ Note that evidence for such homo-coupling defects was found in the UV-VIS spectra above. The increase in mobility is consistent with the higher degree of thin-film order as seen in the GIWAXS data.

As might have been anticipated from the high degree of microstructural order evident in GIWAXS, transistors based on **(l-C_18_)-DPP-(b-C_17_)-BTZ** show the highest FET performance (see Fig. 3a) with high *I*_on_/*I*_off_ ratios of 10^6^ to 10^7^ at low source-drain voltages (*V*_D_ = –5 V), low subthreshold swing values of 1.5 V dec^–1^ and high average saturated hole mobilities of 2.4 cm^2^ V^–1^ s^–1^ with maximum values for hole mobilities ranging up to 2.8 cm^2^ V^–1^ s^–1^. Electron injection from the Au contacts into the **(l-C_18_)-DPP-(b-C_17_)-BTZ** polymer films seems to be slightly hindered as evident from the lack of transfer curve symmetry, especially at low gate voltages (*V*_D_ = –5 V) and by the high relative voltage difference between drain and gate electrode needed to induce n-type transport. It was not possible to operate the transistors in the electron transport regime while using positive source-drain voltages because the high positive gate voltages needed to observe n-type transport using positive *V*_D_ usually lead to a breakdown of the polymer gate dielectric. Nevertheless transistors based on **(l-C_18_)-DPP-(b-C_17_)-BTZ** showed high average saturated electron mobilities around 1.5 cm^2^ V^–1^ s^–1^ with maximum electron mobilities ranging up to 2.4 cm^2^ V^–1^ s^–1^. The mobilities achieved with **(l-C_18_)-DPP-(b-C_17_)-BTZ** for either holes and electrons are amongst the highest values published for DPP-containing polymer semiconductors in top-gated structures.^4^,^6^,^7^,^32^ Even though mobilities exceeding 3 cm^2^ V^–1^ s^–1^ have been published for devices using bottom-gated structures, these devices usually exhibit severe non-idealities in their device characteristics which manifest as kinks in the *I*_D_^1/2^ over *V*_G_ plot. These kinks lead to an unusual gate-voltage dependency of mobilities with very high mobilities in a small region at low *V*_G_ and much lower mobilities at higher *V*_G_ when the transistor is switched on completely.^2^,^22^,^33^ As pointed out in the experimental section our transistors do not show this behaviour and the extracted mobilities are valid in a large region of the transfer curve. As obvious from the transport data, the introduction of a branched side-chain at the BTZ unit as in **(l-C_18_)-DPP-(b-C_17_)-BTZ** improves charge-transport significantly compared to the linear side-chain in **(l-C_18_)-DPP-(l-C_8_)-BTZ**. However it is still surprising that the introduction of a single branched side-chain on the BTZ unit leads to such a dramatic improvement in charge-carrier mobility compared to a linear side-chain. A possible explanation for this is the planarization of the polymer backbone by a more favourable side-chain arrangement leading to an improvement in thin-film order, a more planar backbone conformation with longer conjugation lengths and a reduced reorganization energy. This is consistent with the absorption spectrum of **(l-C_18_)-DPP-(b-C_17_)-BTZ** showing a high 0-0/0-1 absorption peak intensity ratio which is indicative for a low reorganization energy and long conjugation lengths.

Low reorganization energies caused by different structural arrangement due to differences in side-chain packing in **(l-C_18_)-DPP-(b-C_17_)-BTZ** and **(l-C_18_)-DPP-(l-C_8_)-BTZ** can also explain the difference in annealing dependence of the saturation mobilities of the two polymers compared to **(l-C_16_)-DPP-(l-C_8_)-BTZ** and **(b-C_20_)-DPP-(l-C_8_)-BTZ**. Due to higher degrees of film order and therefore low-reorganization energies already being present at low annealing temperatures in **(l-C_18_)-DPP-(b-C_17_)-BTZ** and **(l-C_18_)-DPP-(l-C_8_)-BTZ** higher annealing temperatures do not lead to large enhancements in charge-carrier mobility in these polymers. Even though **(l-C_18_)-DPP-(l-C_8_)-BTZ** and **(l-C_16_)-DPP-(l-C_8_)-BTZ** mainly differ in molecular weight, a small difference in side-chain length and the used synthesis method, the polymers do not show the same annealing behavior. If annealed at temperatures higher than 110 °C the hole mobility of **(l-C_18_)-DPP-(l-C_8_)-BTZ** decreases while the electron mobility steadily increases up to annealing temperatures of 300 °C. **(l-C_18_)-DPP-(b-C_17_)-BTZ** essentially shows the same annealing behavior as **(l-C_18_)-DPP-(l-C_8_)-BTZ**, even though the increase in electron mobility is not as pronounced. In comparison **(l-C_16_)-DPP-(l-C_8_)-BTZ** and **(b-C_20_)-DPP-(l-C_8_)-BTZ** both show a significant increase in saturated hole and electron mobility when annealed at temperatures of up to 300 °C which could be caused by the need to eliminate specific trapping sites and/or to enhance thin-film ordering and therefore lower reorganization energy by annealing at high temperatures for these polymer synthesized by DARP.

As shown by GIWAXS measurement of films of **(l-C_18_)-DPP-(bC_17_)-BTZ** at different annealing temperatures (see Fig. S3†) the semicrystalline order in **(l-C_18_)-DPP-(b-C_17_)-BTZ** increases when annealed at higher temperatures. However no related increase in hole mobilities can be observed suggesting that the film morphology at 110 °C annealing temperature is already ordered enough to enable the highest possible degree of charge-transport performance in spin-coated films of **(l-C_18_)-DPP-(b-C_17_)-BTZ**.

To gain a deeper insight into the charge transport of the studied polymers, temperature dependent measurements were performed on transistors annealed at temperatures ensuring optimal transistor performance (*i.e.* 300 °C for **(l-C_16_)-DPP-(l-C_8_)-BTZ** and **(b-C_20_)-DPP-(l-C_8_)-BTZ** and 110 °C for polymers **(l-C_18_)-DPP-(b-C_17_)-BTZ** and **(l-C_18_)-DPP-(l-C_8_)-BTZ)**.

Fig. S4† shows the dependence of the extracted saturated charge-carrier mobilities of **(l-C_16_)-DPP-(l-C_8_)-BTZ** and **(b-C_20_)-DPP-(l-C_8_)-BTZ** as a function of measurement temperature. The carrier mobilities show a simple Arrhenius-like, temperature activated behavior as demonstrated by the linear dependence of mobility on a 1/T scale. Activation energies for holes and electrons of 135 meV and 141 meV for **(l-C_16_)-DPP-(l-C_8_)-BTZ** and 157 meV and 214 meV for **(b-C_20_)-DPP-(l-C_8_)-BTZ** can be extracted. The decreased activation energy for charge-carrier hopping in **(l-C_16_)-DPP-(l-C_8_)-BTZ** in comparison with **(b-C_20_)-DPP-(l-C_8_)-BTZ** is consistent with the increased charge-carrier mobilities. As expected from the increased mobility and the higher degree of structural order, the activation energy for charge-transport in **(l-C_18_)-DPP-(l-C_8_)-BTZ** with values of 100 meV for holes and electrons is even smaller than for **(l-C_16_)-DPP-(l-C_8_)-BTZ**.

**Fig. 5:**
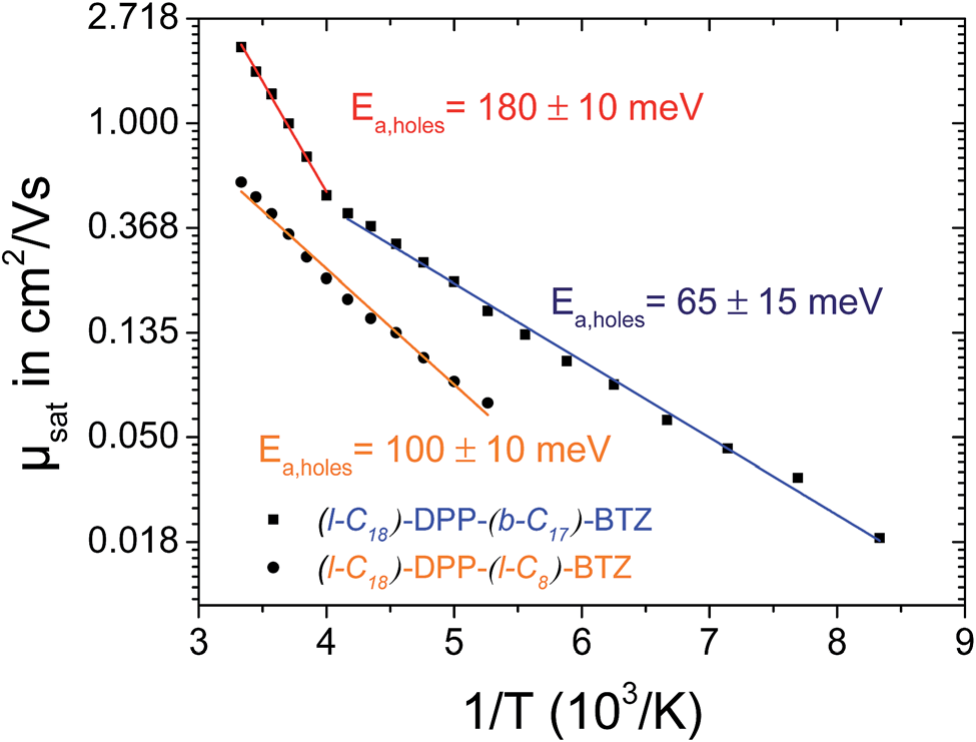
Arrhenius plot of temperature dependent p-type field effect mobility of **(l-C_18_)-DPP-(b-C_17_)-BTZ** (squares) and **(l-C_18_)-DPP-(l-C_8_)-BTZ** (circles). The blue, the red and the orange curves show linear fits for the activation energy extraction in the high and low temperature region (above and below 250 K) for **(l-C_18_)-DPP-(b-C_17_)-BTZ** (red/blue) and across the whole temperature range for **(l-C_18_)-DPP-(l-C_8_)-BTZ** (orange). All measurements were taken in forward and reverse direction to.

In contrast to **(l-C_18_)-DPP-(l-C_8_)-BTZ**, the low-temperature charge transport measurements of **(l-C_18_)-DPP-(b-C_17_)-BTZ** do not show a simple Arrhenius behavior with just one slope. The Arrhenius plot of saturated hole-mobility over 1/T shown in Fig. 5 shows two clearly distinguishable regions with different slopes in the temperature regime ranging from 300 down to 120 K with a change of temperature behavior at around 250 K for **(l-C_18_)-DPP-(b-C_17_)-BTZ**. In the high temperature region above 250 K a high activation energy of 180 meV is extracted, while in the low temperature region below 250 K a much lower activation energy value of around 65 meV is observed suggesting different charge-transport mechanisms in the two regimes. The charge-transport activation energy value of 180 meV extracted from the high temperature regime is unusually high for a high mobility polymer, as other high mobility polymers like PBTTT, DPP-BT or PSeDPP-BT usually show much lower activation energies in the range of 50–90 meV.^4^,^34^,^35^

A similar temperature dependent transport behavior with two distinct activation energy regimes was already proposed by Street *et al.* in 2005 for semicrystalline films of PQT-12.^36^ In this publication Street *et al.* explained the two different activation energy regions by the activation of charge transport through the disordered grain boundary regions at higher temperatures leading to an increase in activation energy. Recently Noriega *et al.* extended this model to explain the exceptionally high charge-carrier mobility values of poorly ordered polymers like DPP-BT by intrachain transport along long polymer tie-chains bridging the disordered regions between small semicrystalline domains.^35^ A comparable model based on percolation transport model can be used to explain the observed temperature dependence of charge-transport activation energy in **(l-C_18_)-DPP-(b-C_17_)-BTZ**.

We propose that charge-transport in **(l-C_18_)-DPP-(b-C_17_)-BTZ** at temperatures below 250 K is dominated by a few percolation paths leading through more ordered regions of the film and mainly containing low-activation energy hops. At temperatures above 250 K the higher thermal energy enables additional transport paths to occur through somewhat more disordered regions of the polymer thin film increasing the measurable activation energy to values close to activation energies usually observed in purely amorphous polymers like regioregular-amorphous-P3HT.^37^ Interestingly the same behaviour is not observed in **(l-C_18_)-DPP-(l-C_8_)-BTZ** suggesting that in this polymer the difference in energy between the few percolation paths that allow charge transport at low temperature and the transport paths through more disordered regions is too large for the latter to become thermally accessible near room temperature.

## Conclusions

We prepared four new low band-gap DPP-based co-polymers with BTZ to allow for additional solubilizing side-chains on the BTZ co-monomer. We used two different synthetic routes, direct arylation and Suzuki-polycondensation, and found from a comparison of polymers with only linear side chains made by the two routes that direct arylation produces a higher density of chain defects which manifest themselves as low band gap trap sites in UVVIS spectroscopy and lead to a lower electrical performance. We showed that the introduction of a linear side-chain on the DPP-unit leads to an increase in thin-film order and charge-carrier mobility provided that the BTZ co-monomer is substituted with a sufficiently solubilizing branched side chain. **(l-C_18_)-DPP-(b-C_17_)-BTZ** exhibits near balanced electron and hole mobilities; the average hole mobility of 2.4 cm^2^ V^–1^ s^–1^ is exceptionally high, in fact it is one of the highest, robustly extracted values reported for a DPP copolymer in a top-gate configuration to date. Our results demonstrate clearly that linear side chain substitution of the DPP unit is an attractive route for improving the charge transport properties of DPP copolymers. It requires the use of co-monomers however, that allow attachment of sufficiently long or branched side chains to retain sufficient solubility for solution processing. The experimental study presented here demonstrates very clearly that for a given conjugated polymer backbone judicious selection of the side chain substitution is crucial to ensure optimum device performance.

One of the challenges from a practical design point of view is that it is difficult to predict from mere reasoning or simple molecular modelling how a particular side chain substitution is likely to influence the polymer microstructure in the solid state. A microscopic understanding of the sensitive dependence of microstructure on side chain substitution generally requires sophisticated molecular dynamics modelling that goes beyond the scope of the present work.^10^,^38^ However, as such detailed calculations will be performed in the future on more high mobility polymers, such as the ones reported here, general molecular design guidelines for side chain substitution are likely to emerge. Our work suggests that copolymers with linear side chains on the DPP unit and branched side chains on the co-monomer may be an attractive design motif for high mobility DPP copolymers.

## Supplementary Material

Supplementary information
