# Supplementary material for: Enabling high-mobility, ambipolar charge-transport in a DPP-benzotriazole copolymer by side-chain engineering
Source: Chem Sci. 2015 Aug 12;6(12):6949–60. doi: 10.1039/c5sc01326g (PMC5951104; doi:10.1039/c5sc01326g)
Supplement: Supplementary file 1 [file SC-006-C5SC01326G-s001.pdf]

Electronic Supplementary Material (ESI) for Chemical Science.
This journal is © The Royal Society of Chemistry 2015

Supporting Information:

Enabling high-mobility, ambipolar charge-transport in a DPP-Benzotriazole copolymer by side-chain engineering

Mathias Gruber,[†a] Seok-Heon Jung,[†b] Sam Schott,[a] Deepak Venkateshvaran,[a] Auke Jisk Kronemeijer,[a] Jens Wenzel Andreasen,[c] Christopher R McNeill,[d] Wallace W. H. Wong,[e] Munazza Shahid,[f] Martin Heeney,[f] Jin-Kyun Lee[b] and Henning Sirringhaus[a]

Material NMR and Synthesis

Raw materials and solvents

K_2CO_3, Na_2CO_3,18-crown-6, KHF_2 (3 M solution in water), phenylboronic acid, bromobenzene, $Pd(PPh_3)_4$,$Pd(dppf)Cl_2 \cdot$DCM complex were purchased from Sigma-Aldrich and used as received. Dioxane (inhibitor-free, anhydrous), toluene (anhydrous), DMF (anhydrous), were received from Sigma-Aldrich and used without further drying.

NMR

^{1}H NMR spectra were recorded on a Varian Inova-400 (400 MHz) spectrometer at roomtemperature using the chemical shift of the residual protic solvent ($CHCl_3$ at δ 7.24 ppm and DMSO at δ 2.50 ppm) as the internal reference. All chemical shifts are quoted in parts per million (ppm) relative to the internal reference and the coupling constants *J* are measured in Hz. The multiplicity of the signal is indicated as follows: s (singlet), d (doublet), t (triplet), q (quartet), m (multiplet), and br s (broad singlet). ^{13}C NMR spectra were recorded on a Varian Inova-400 (101 MHz) spectrometer using the central resonance of the triplet of $CDCl_3$ at δ 77.0 ppm or the septet of DMSO-d6 at δ 40.0 ppm as a reference. ^{19}F NMR spectra were recorded on a Varian Inova-400 (376 MHz) spectrometer at room temperature.

[a.] *Cavendish Laboratory, University of Cambridge, J J Thomson Avenue, Cambridge, CB3 0HE (United Kingdom). E-mail: hs220@cam.ac.uk*
[b.] *Department of Polymer Science & Engineering, Inha University, Incheon, 402-751 (South Korea). E-mail: jkl36@inha.ac.kr*
[c.] *Department of Energy Conversion and Storage, Technical University of Denmark, Frederiksborgvej 399, 4000 Roskilde (Denmark).*
[d.] *Department of Materials Engineering, Monash University, Clayton, Victoria 3800 (Australia).*
[e.] *School of Chemistry, Bio21 Institute, University of Melbourne, Parkville, Victoria 3010, (Australia).*
[f.] *Department of Chemistry and Centre for Plastic Electronics, Imperial College, London, SW7 2AZ (United Kingdom). E-mail: m.heeney@imperial.ac.uk*
† These authors contributed equally to this work. M. G. designed and performed the device fabrication and polymer characterization experiments, analysed the experimental data and wrote the paper while S-H. J. synthesized the polymers (l-C_{18})-DPP-(b-C_{17})-BTZ and (l-C_{18})-DPP-(l-C_8)-BTZ and wrote the polymer synthesis sections.
DOI: 10.1039/x0xx00000x

Synthesis

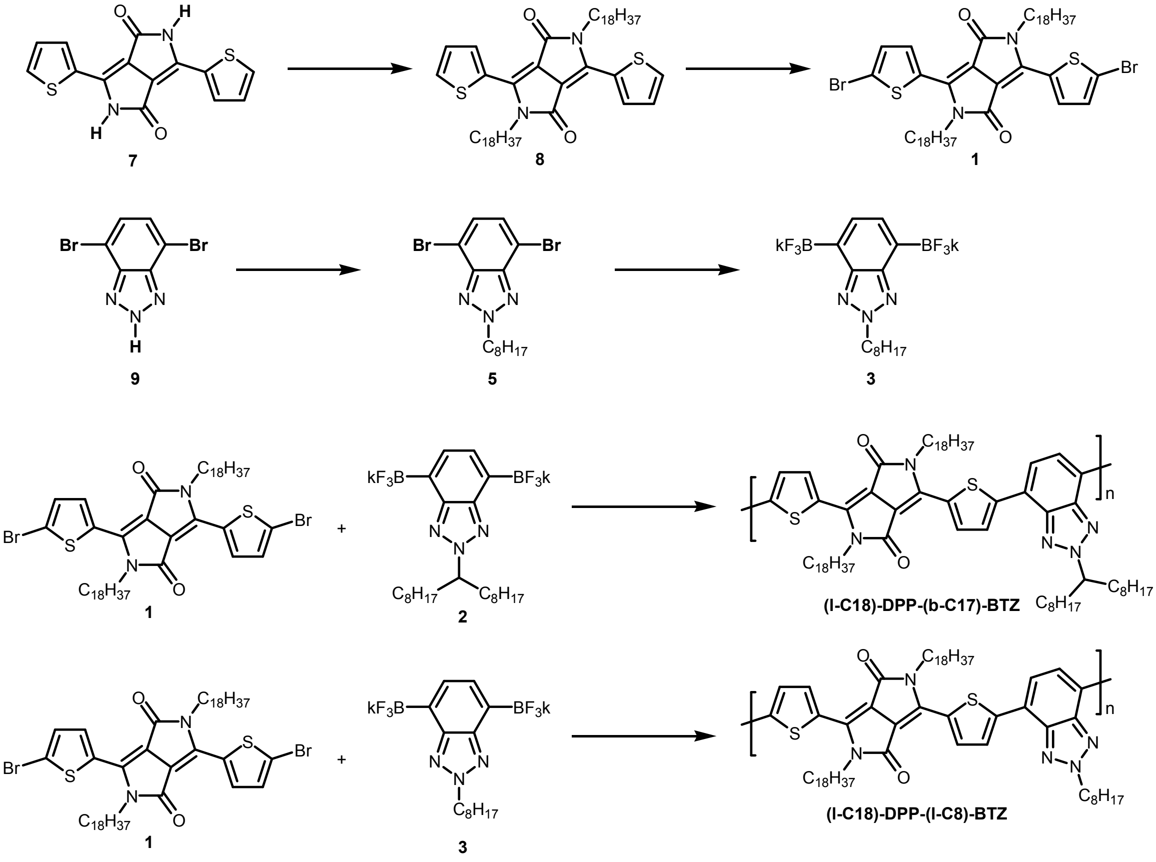

Scheme S 1. Synthesis of monomers and polymers.

2,5-dioctadecyl-3,6-di(thiophen-2-yl)pyrrolo[3,4-c]pyrrole-1,4(*2H,5H*)-dione [**(l-C18)-DPP**, **8**]

A round-bottomed flask was filled with 3,6-di(thiophen-2-yl)pyrrole[3,4-c]pyrrole-1,4(*2H-5H*)-dione **7** (5.00 g, 16.6 mmol),[1] K_2CO_3 (9.2 g, 67 mmol), 18-crown-6 (0.05 g), and DMF (anhydrous, 70 cm^3). The reaction mixture was heated at 130 °C under N_2 atmosphere. 1-Bromooctadecane (17 g, 50 mmol) was added to the mixture by syringe. After the reaction was stirred for another 20 h at 130 °C, the solution was cooled to room temperature and poured into the ice-water mixture (200 cm^3). The precipitate was filtered and rinsed with a copious amount of water. The filter cake was washed further with MeOH several times. The crude product was purified by column chromatography (silica gel, $CHCl_3$) to give dark red **(l-C18)-DPP** (4.1 g, 31%); ^{1}H NMR (400 MHz, $CDCl_3$, δ) 8.91 (2H, dd, J = 4 Hz, 1 Hz, Ar-H), 7.26 (2H, dd, J = 5 Hz, 1Hz, Ar-H), 4.04 (4H, t, J= 8 Hz, NCH_2), 1.72 (4H, m), 1.50 - 1.20(60H, m), 0. 85 (6H, t, J= 6 Hz, CH_3) The δ_H data was in agreement with the literature values.[2]

3,6-bis(5-bromothiophene-2-yl)-2,5-dioctadecylpyrrolo[3,4-c]pyrrole-1,4(*2H,5H*)-dione [**(l-C18)-DPP dibromide**, **1**]

To a magnetically stirred solution of **(l-C18)-DPP 8** (1.80 g, 2.24 mmol) was dissolved into $CHCl_3$ (30 cm^3). Br_2 (0.75 g, 4.7 mmol) dissolved in $CHCl_3$ (1.5 cm^3) was added to the reaction vessel at room temperature. The mixture was stirred at room temperature for 30 min and then heated to 60 °C. After 2 h, the solution was cooled to room temperature and the precipitate was recovered by filtration. The filter cake was the washed with Na_2SO_3 solution, water, and methanol several times. The crude product was purified by column chromatography (silica gel, $CHCl_3$) and then crystallized from $CHCl_3$ to give dark blue **(l-C18)-DPP dibromide** (1.8 g, 84%); ^{1}H NMR (400 MHz, $CDCl_3$, δ) 8.67 (2H, d, J = 4 Hz, Ar-H), 7.24 (2H, t, J = 4 Hz, Ar-H), 3.97 (4H, t, J= 8 Hz, NCH_2), 1.70 (4H, m), 1.45 - 1.20(60H, m), 6.87 (6H, t, J= 7 Hz, CH_3) The δ_H data was in agreement with the literature values.[2]

4,7-dibromo-2-octyl-*2H*-benzo[*d*][1,2,3]triazole [**(l-C8)-BTZ dibromide**, **5**]

A 250 cm^3 round-bottomed flask was filled with the 4,7-dibromobenzotriazole (9.00 g, 32.5 mmol) and K_2CO_3 (8.9 g, 65 mmol). DMF (anhydrous, 80 cm^3) was added to the flask and the solution was heated to 90 °C. 1-Bromooctane(6.9 g, 36 mmol) was added in one portion by syringe. After the reaction mixture was stirred at 90 °C for 2 h, the solution was cooled to room temperature and quenched by the addition of water (120 cm^3). The product was extracted with Et_2O (120 cm^3). The organic layer was washed with brine (100 cm^3), dried over anhydrous $MgSO_4$, and concentrated under reduced pressure. The crude product was purified by column chromatography (silica gel, CH_2Cl_2:hexane = 1:3 by vol.) and then crystallized from an isopropanol/EtOAc mixture to give colourless crystalline **(l-C8)-BTZ dibromide** (8.2 g, 65%); ^{1}H NMR (400 MHz, $CDCl_3$, δ) 7.44 (2H, s, Ar-H), 4.75 (2H, t, J = 7.5 Hz, NCH_2), 2.15 - 2.10 (2H, m, $NCH_2\underline{CH_2}$), 1.35 - 1.25 (10H, m). 0.86(3H, t, J = 7.0, CH_3) The δ_H data was in agreement with the literature values.[3]

Dipotassium [2-octyl-*2H*-benzotriazole]-4,7-bis(trifluoroborate) [**dipotassium (l-C8)-BTZ bis(trifluoroborate)**, **3**]

To a Schlenk tube were added **(l-C8)-BTZ dibromide 3** (1.00 g, 2.57 mmol), KOAc (1.51 g, 15.2 mmol), bis(pinacolato)diboron (1.96 g, 7.71 mmol), and Pd(dppf)Cl_2·DCM complex (0.10 g, 0.13 mmol). The tube was purged with N_2. Dioxane (N_2 bubbled, 20 cm^3) was added to the reaction vessel and solution was stirred at 90 °C for 1 h. The reaction mixture was

cooled to room temperature. The mixture was poured into ice-water (100 cm^3) and the product was extracted with Et_2O (150 cm^3). The organic layer was washed with brine (100 cm^3), dried over anhydrous $MgSO_4$, and concentrated under reduced pressure. The resulting viscous liquid was diluted with MeOH (60 cm^3) at room temperature. An aqueous solution of KHF_2 (3 M, 11 cm^3, 32 mmol) was added to the MeOH solution, resulting in the precipitation of solid product. After the suspension was stirred for 1 h, the solid was recovered by filtration and washed with a small amount of water. The crude product was purified by recrystallization from an acetonitrile/water mixture twice to give **dipotassium (l-C8)-BTZ bis(trifluoroborate)** as colourless crystals (0.68 g, 79%); (Found: C, 38.03; H, 4.36; N, 9.42. $C_{14}H_{19}H_2F_6K_2N_3$ requires C, 37.95; H, 4.32; N, 9.48%) ^{1}H NMR (400 MHz, DMSO-d_6, δ) 7.04 (2H, s, Ar-H), 4.55 (2H, t, J = 7.5 Hz, NCH_2), 1.94 - 1.86 (2H, m, $NCH_2\underline{CH_2}$), 1.28 - 1.21 (10H, m). 0.81(3H, t, J = 7.0, CH_3) ^{13}C NMR (100 MHz, DMSO-d_6, δ) 147.31, 128.20, 55.62, 31.91, 30.76, 29.38, 29.32, 26.96, 22.73, 14.60 ppm; ^{19}F NMR (376 MHz, DMSO-d_6, δ) -130.6 ppm(d, J = 18 Hz).

TGA and DSC

Thermogravimetric analyses (TGA) were performed on a NETZSCH TG 209 F3 thermogravimetric analyzer at a heating rate of 10 °C min-1 under a N2 atmosphere. Thermal transition behaviours of polymers were measured on a NETZSCH DSC 404 F1 modulated differential scanning calorimeter (DSC) at a heat/cool rate of 10 °C min-1 under N2 for three heat/cool cycles.

No appreciable glass transitions were detected in any of the polymers.

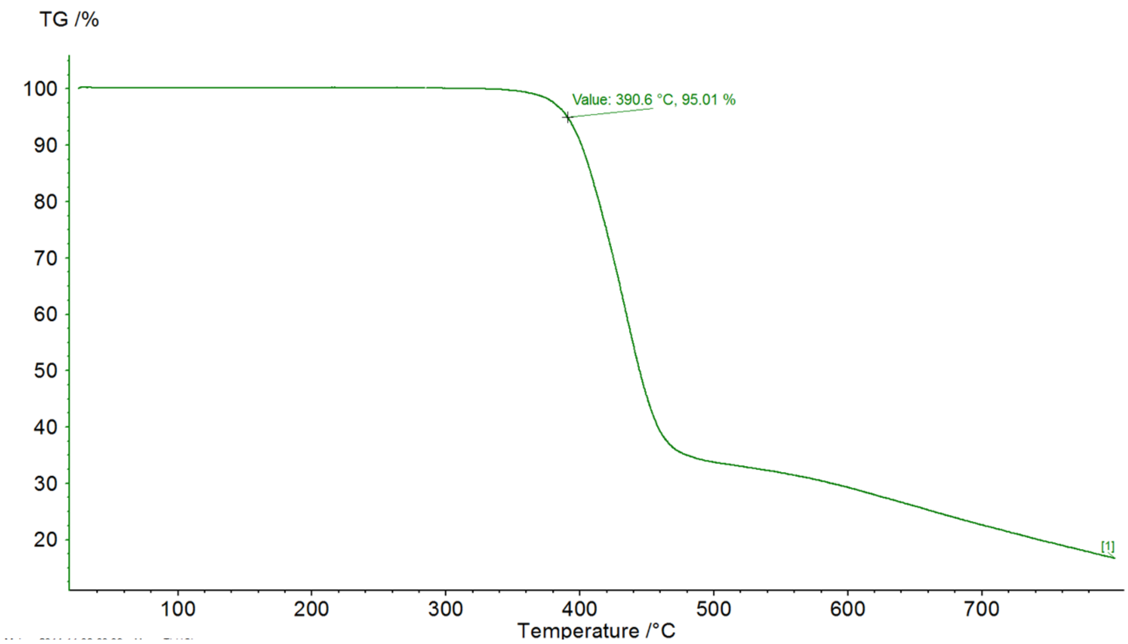

Figure S 1. TGA thermogram of **(l-C18)-DPP-(b-C17)-BTZ**.

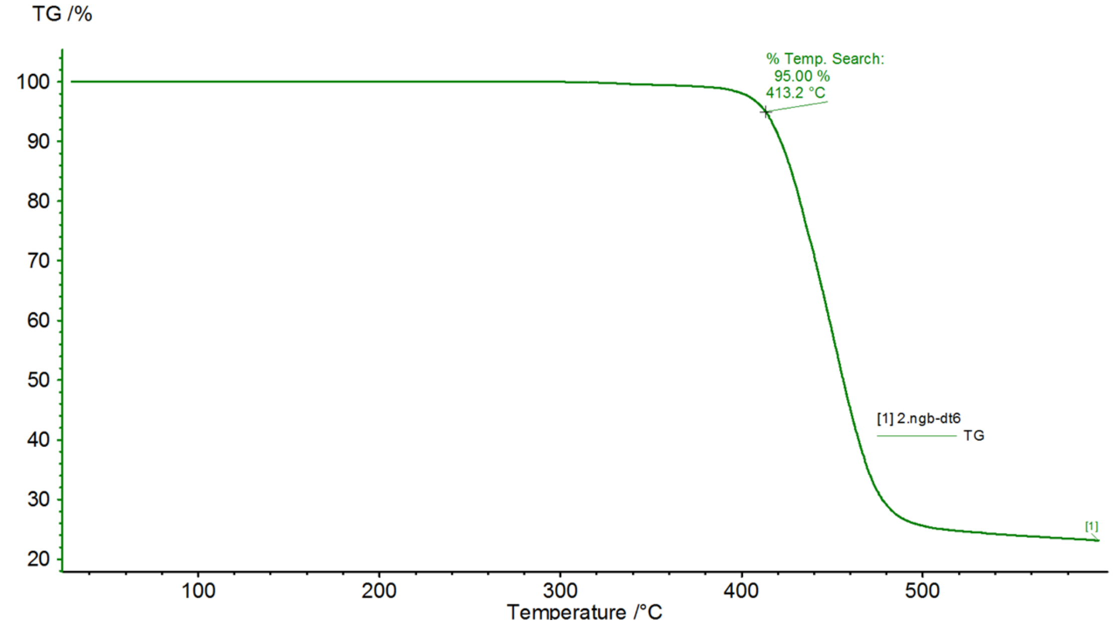

Figure S 2. TGA thermogram of **(l-C18)-DPP-(l-C8)-BTZ**.

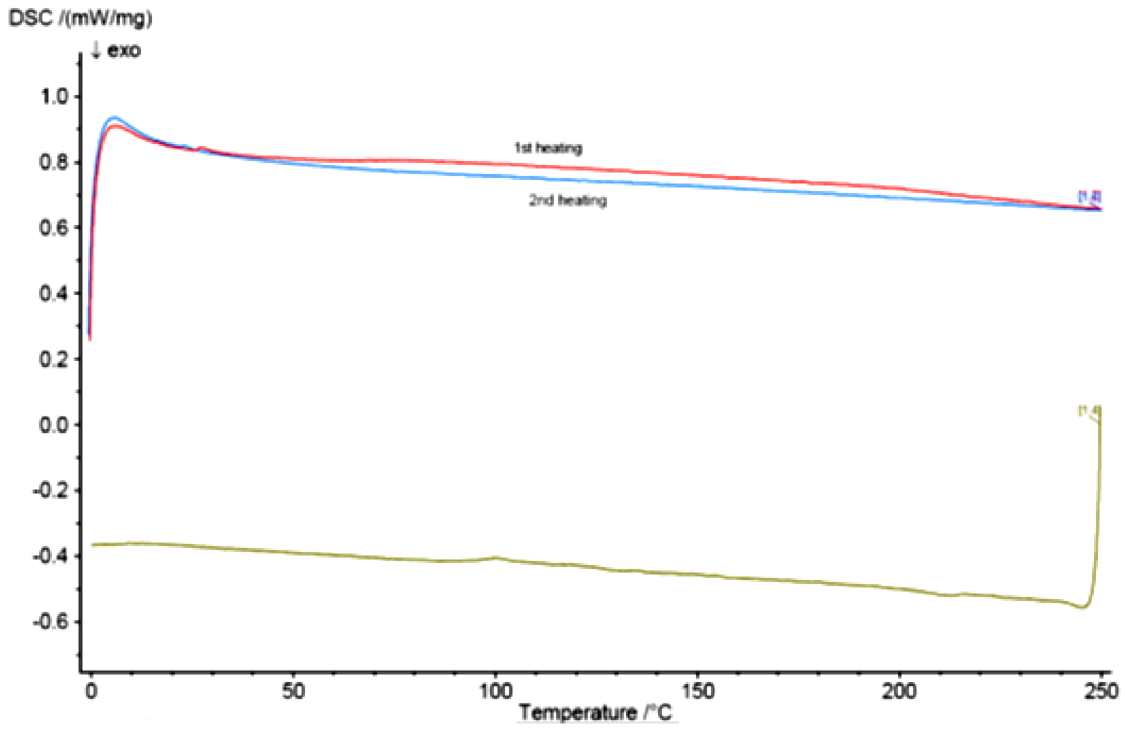

Figure S 3. DSC thermogram of **(l-C18)-DPP-(b-C17)-BTZ.**

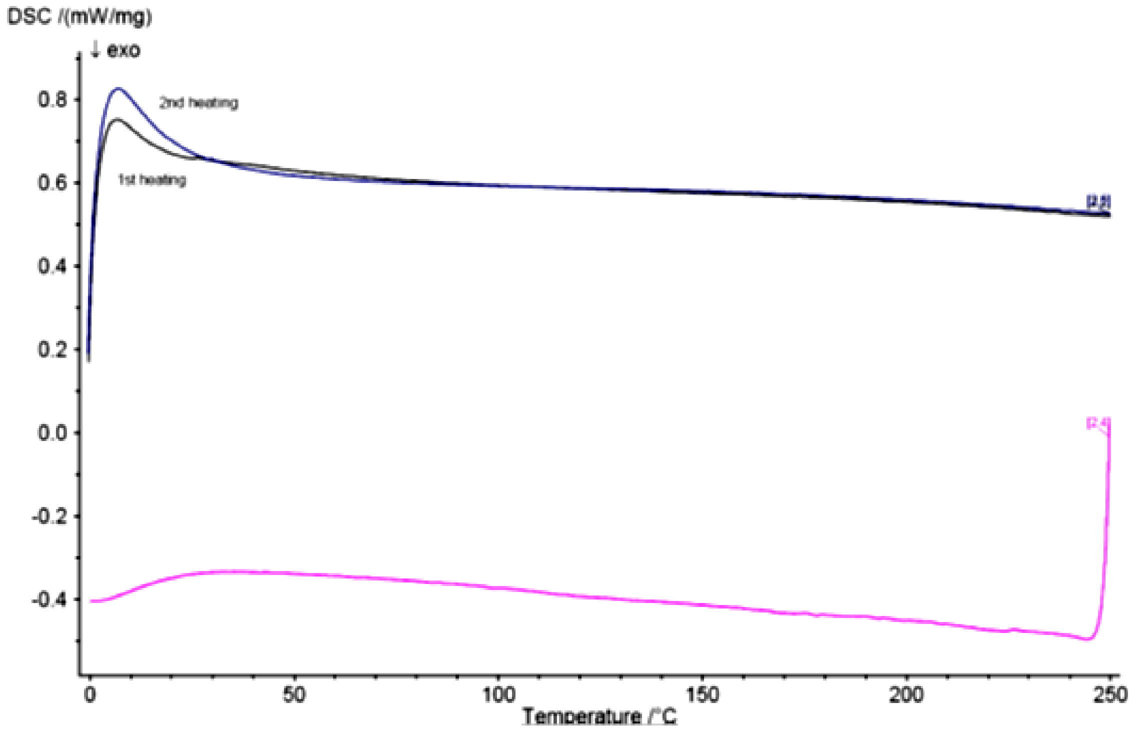

Figure S 4. DSC thermogram of **(l-C18)-DPP-(l-C8)-BTZ.**

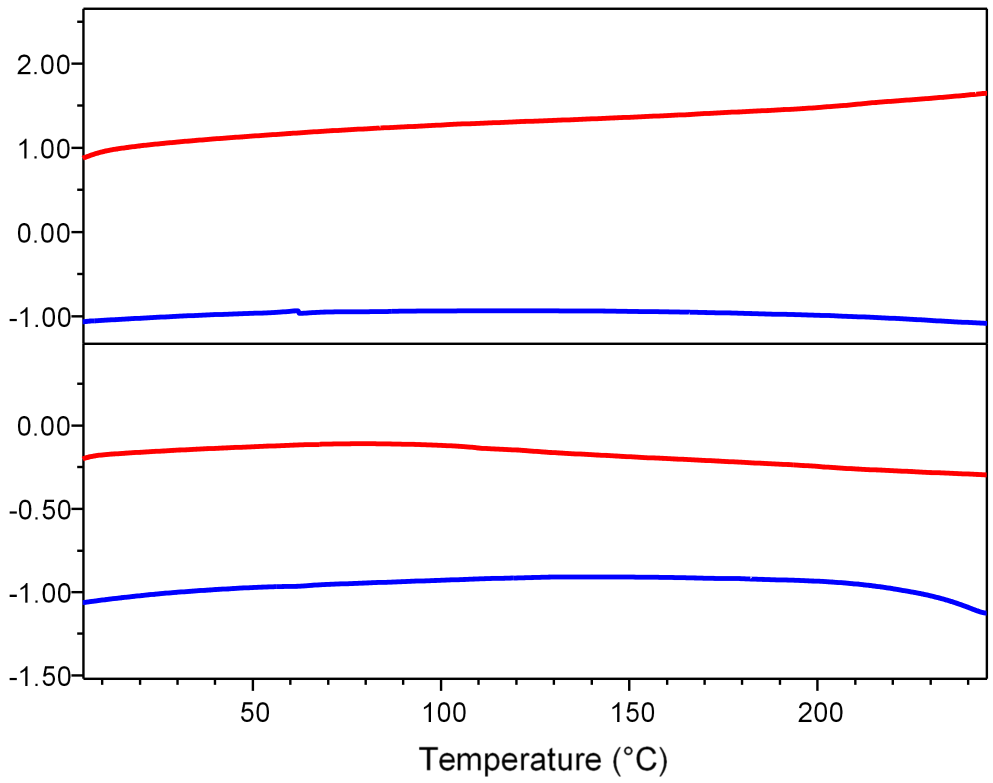

Figure S 5. DSC thermogram of **(1-C16)-DPP-(l-C8)-BTZ** (top) and **(b-C20)-DPP-(l-C8)-BTZ** (bottom) at 10°C min heating and cooling rate (second cycle shown, heating trace (red), cooling trace (blue)).

Gel permeation chromatography (GPC)

Gel permeation chromatography for (b-C20)-DPP-(l-C8)-BTZ and (l-C16)-DPP-(l-C8)-BTZ were performed on a Agilent Technologies 1200 series GPC by eluting chlorobenzene at 80 °C, using two PL mixed B columns in series, and calibrated against narrow polydispersity polystyrene standards.

Cyclic voltametry

Cyclic voltammetry (CV) experiments for **(l-C18)-DPP-(b-C17)-BTZ** and **(l-C18)-DPP-(l-C8)-BTZ** were performed at a sweep rate of 50 mV/s. CVs were carried out in a three-electrode cell consisting of a glassy carbon working electrode, a platinum wire auxiliary electrode, and a Ag/Ag+ pseudo-reference electrode. The supporting electrolyte was 0.10 M tetrabutylammonium hexafluorophosphate (Bu4NPF6) in CH3CN. The solutions were deoxygenated by sparging with argon prior to each scan and blanketed with argon during the scans. The glassy carbon working electrode was prepared by polishing with 5 μm alumina and washed and dried before the polymer was drop-casted on the electrode from chlorobenzene solution to form a film. Ferrocene/ferrocenium redox couple was used as the internal standard. The HOMO energy level was calculated from the onset of the oxidation potential of the polymer using the following: EHOMO = - (4.8 + Eox onset) eV.

Table of electrochemical data

Polymer	Oxidation onset (V)	Reduction onset (V)	Abs onset (nm)	Optical band gap (eV)	E_{HOMO} (eV)	E_{LUMO} (eV)
P3HT	0.10	-	650	1.91	-4.90	-2.99
**(l-C18)-DPP-(l-C8)-BTZ**	0.33	-1.50	940	1.32	-5.13	-3.81
**(l-C18)-DPP-(b-C17)-BTZ**	0.35	-1.55	990	1.25	-5.15	-3.90

Note that E_{LUMO} is calculated from E_{HOMO} and the optical band gap. The reduction onset values should not be used to estimate E_{LUMO} as the CV of the reduction process was not reversible.

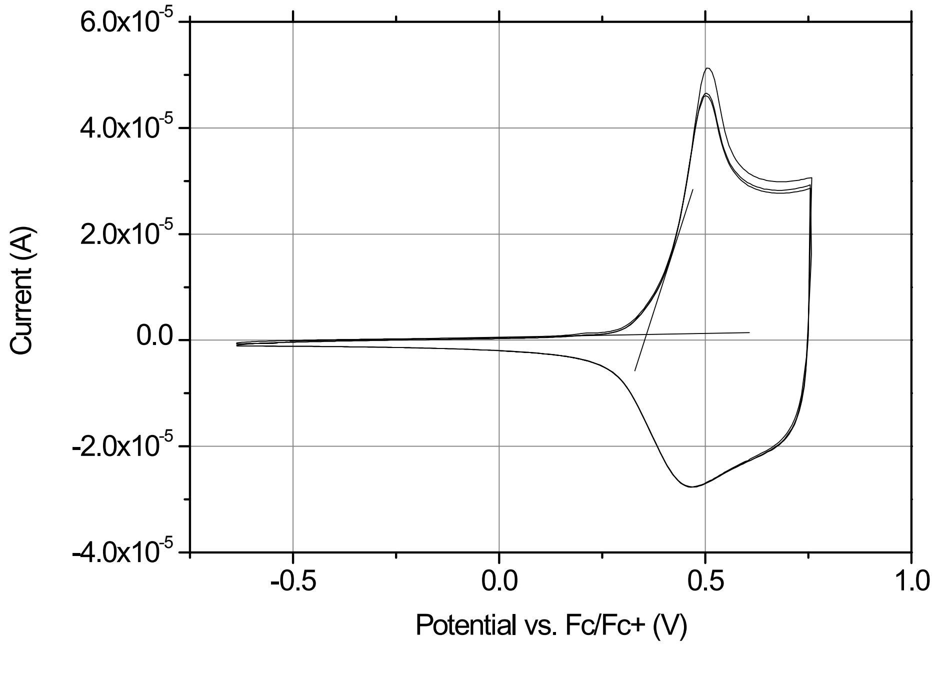

Cyclic voltammogram of **(l-C_{18})-DPP-(b-C_{17})-BTZ**.

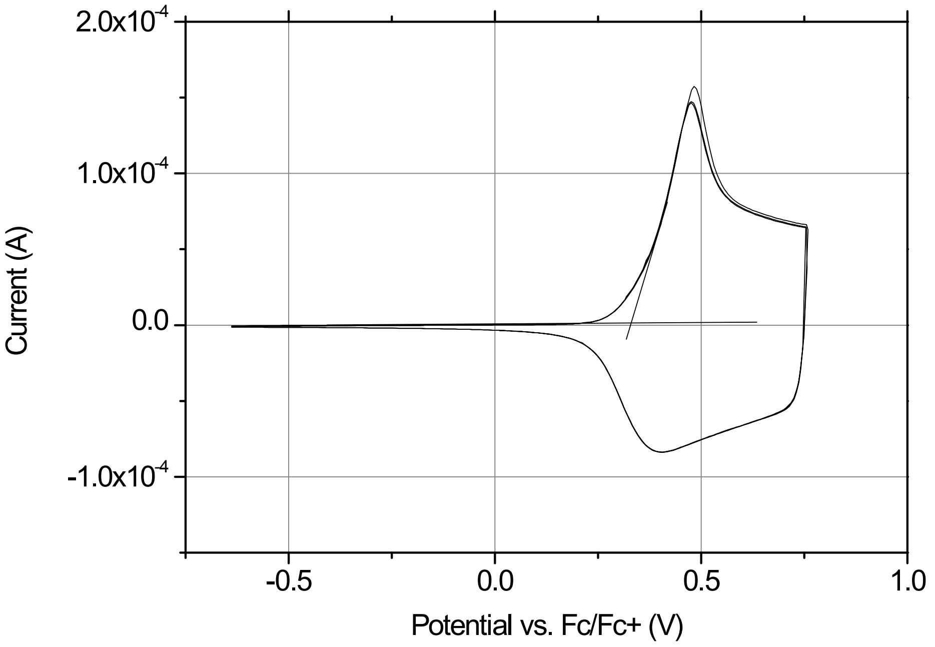

Cyclic voltammogram of **(l-C$_{18}$)-DPP-(l-C$_{8}$)-BTZ**.

GIWAXS structural characterization

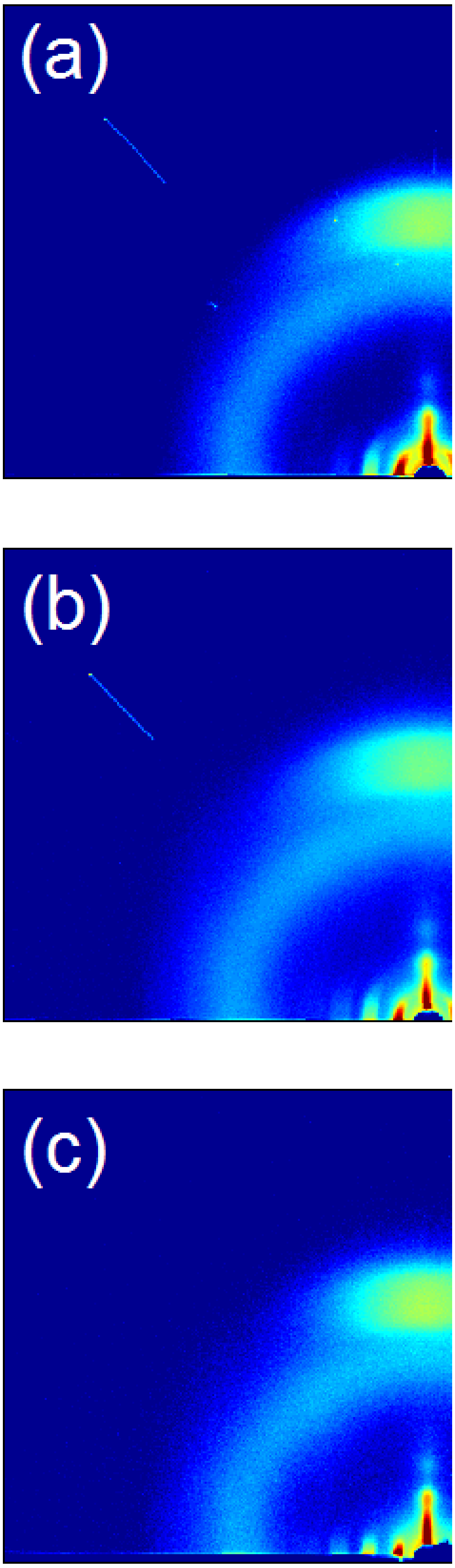

Figure S 6: 2D GIWAXS patterns of ***(l-C_{18})*-DPP-*(b-C_{17})*-BTZ** films annealed at **(a)** 110 °C, **(b)** 200 °C and **(c)** 300 °C.

Temperature dependent charge-transport

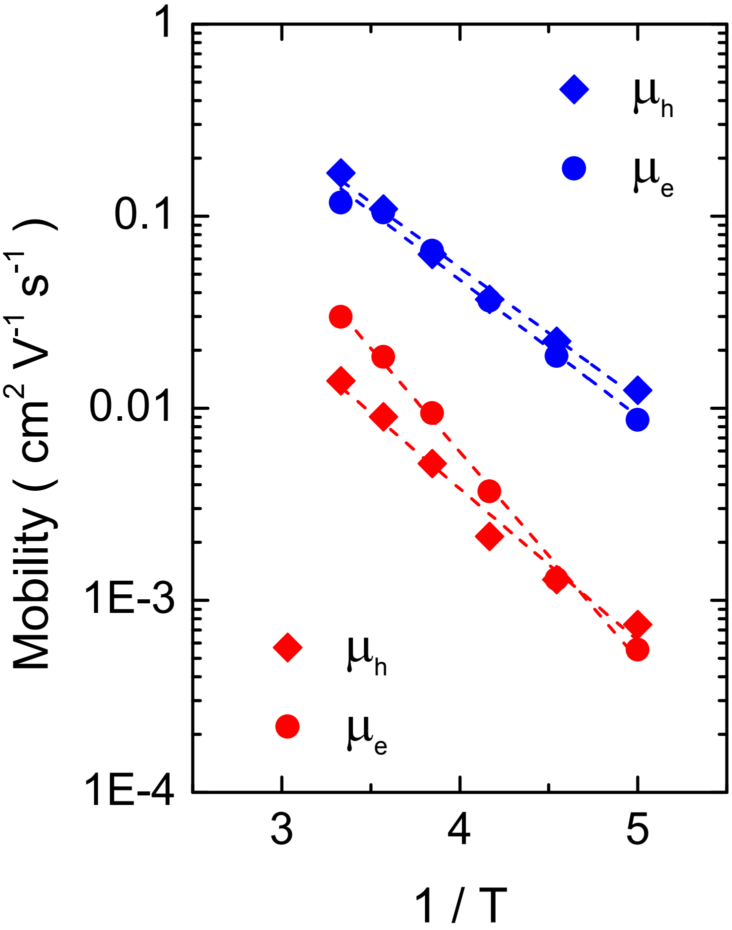

Figure S 7: Arrhenius plot of temperature dependent p-type field effect mobility of **(b-C_{20})-DPP-(l-C_8)-BTZ** (red) and **(l-C_{16})-DPP-(l-C_8)-BTZ** (blue). Lines are fitlines used to extract charge-carrier hopping activation energies.

References

[1]L. T. Dou, J. Gao, E. Richard, J. B. You, C. C. Chen, K. C. Cha, Y. J. He, G. Li, Y. Yang, *J. Am. Chem. Soc.* **2012**, *134*, 10071.
[2]B. Y. Fu, J. Baltazar, A. R. Sankar, P. H. Chu, S. Y. Zhang, D. M. Collard, E. Reichmanis, *Adv. Funct. Mater.* **2014**, *24*, 3734.
[3]T. Nakanishi, Y. Shirai, T. Yasuda, L. Y. Han, *J. Polym. Sci,. Part A*. **2012**, *50*, 4829.
